# Metallocene-Naphthalimide Derivatives: The Effect of Geometry, DFT Methodology, and Transition Metals on Absorption Spectra

**DOI:** 10.3390/molecules28083565

**Published:** 2023-04-19

**Authors:** Christina Eleftheria Tzeliou, Demeter Tzeli

**Affiliations:** 1 Laboratory of Physical Chemistry, Department of Chemistry, National and Kapodistrian University of Athens, Panepistimiopolis Zografou, 15784 Athens, Greece; ctzeliou@chem.uoa.gr; 2 Theoretical and Physical Chemistry Institute, National Hellenic Research Foundation, 48 Vassileos Constantinou Ave., 11635 Athens, Greece

**Keywords:** DFT calculations, metallocene, 4-amino-1,8-naphthalimide, piperazine, absorption spectra, ferrocene, cobaltocene, nickelocene

## Abstract

In the present paper, the photophysical properties of metallocene-4-amino-1,8-naphthalimide-piperazine molecules (**1-M^2+^**), as well as their oxidized and protonated derivatives (**1−M^3+^**, **1-M^2+^-H^+^**, and **1-M^3+^-H^+^**), where M = Fe, Co, and Ni, were studied via DFT and TD-DFT, employing three functionals, i.e., PBE0, TPSSh, and wB97XD. The effect of the substitution of the transition metal M on their oxidation state, and/or the protonation of the molecules, was investigated. The present calculated systems have not been investigated before and, except for the data regarding their photophysical properties, the present study provides important information regarding the effect of geometry and of DFT methodology on absorption spectra. It was found that small differences in geometry, specifically in the geometry of N atoms, reflect significant differences in absorption spectra. The common differences in spectra due to the use of different functionals can be significantly increased when the functionals predict minima even with small geometry differences. For most of the calculated molecules, the main absorption peaks in visible and near-UV areas correspond mainly to charge transfer excitations. The Fe complexes present larger oxidation energies at 5.4 eV, whereas Co and Ni complexes have smaller ones, at about 3.5 eV. There are many intense UV absorption peaks with excitation energies similar to their oxidation energies, showing that the emission from these excited states can be antagonistic to their oxidation. Regarding the use of functionals, the inclusion of dispersion corrections does not affect the geometry, and consequently the absorption spectra, of the present calculated molecular systems. For certain applications, where there is a need for a redox molecular system including metallocene, the oxidation energies could be lowered significantly, to about 40%, with the replacement of the iron with cobalt or nickel. Finally, the present molecular system, using cobalt as the transition metal, has the potential to be used as a sensor.

## 1. Introduction

The development of artificial receptors for the sensing and recognition of species, as well as for advanced logic functions, is a significant challenge in the field of molecular information technology [1,2,3,4]. Molecules respond to changes in their environment, i.e., the presence of cations, anions or neutral species, pH, temperature, viscosity, etc., resulting in the alteration of absorption spectra (change of color) or fluorescence spectra (change of intensity or band shifts) [1,2,3,4,5,6,7,8,9,10]. These changes are affected by photoinduced electron transfer (PET), internal charge transfer (ICT), electronic energy transfer (EET), proton transfer (PT), and photochromic processes (PC) [1,2,4,8,10].

Chemosensors are molecules that are used for the sensing of an analyte to produce a detectable change or a signal [11,12,13,14,15]. Their action relies on an interaction occurring at the molecular level, usually involving the continuous monitoring of the activity of a chemical species in a given matrix-like solution, air, blood, tissue, waste effluents, or drinking water. Specifically, sensors presenting changes in their optical properties, i.e., absorption and emission spectra, are preferable.

The development of sensors and molecular logic gates (MLG) responsive to oxidizability and acidity that incorporate a ferrocene (Fc) moiety and bioactive fluorophores have attracted research interest [16,17,18]. For instance, compounds which are AND ([16] and references therein) and INHIBIT [19,20,21,22] MLG with two inputs (H^+^ and Fe^3+^) have been synthesized. Furthermore, three-input AND [17,18,23] and AND-INHIBIT-OR [24] MLG, which can be used as a lab-on-a-molecule have been developed [25]. Recently, we studied, theoretically, via DFT/TD-DFT calculations, the photophysical properties of a three-input AND molecular logic gate [18], which presents an enhanced fluorescence spectrum [17,18] and it had been synthesized by Magri et al. [17] Its properties were explained, and we concluded that molecular systems with N atoms, whose geometry is between planar and tetrahedral, can be ideal molecules for use as sensors and molecular logic gates [18]. Our calculated absorption and emission spectra were in excellent agreement with available experimental data from Magri et al. [17].

In the last two decades, extensive research has been conducted on developing new donor–acceptor molecular systems. An attractive electrondeficient moiety that has been widely used is the 1,8-naphthalimide group [26,27]. Derivatives of this group have various technological applications, such as in pH sensors [28], metal sensors [29], laser dyes [30], fluorescent dyes [31], organic photovoltaics (OPVs) [32], organic light-emitting diodes (OLEDs) [33], bioimaging [34]. The functionalization of 1,8-naphthalimide is cost effective and it easily allows the production of derivatives with tunable photophysical and electronic properties [26,27,28,29,30,31,32,33,34,35,36,37,38,39,40].

**Scheme 1:**
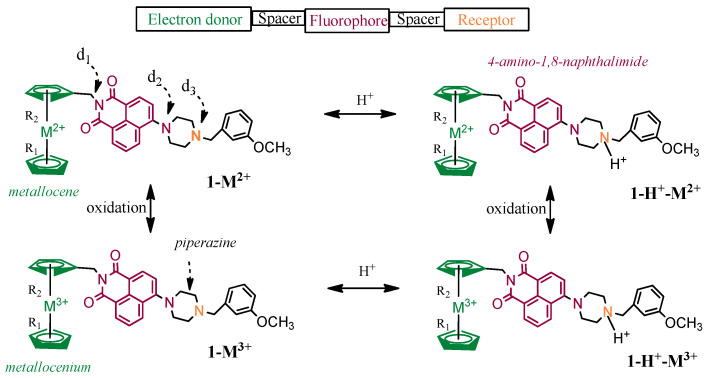
Calculated **1-M^2+^**, **1-M^3+^**, **1-M^2+^-H^+^**, and **1-M^3+^-H^+^** molecular structures.

In the present study, the photophysical properties of molecules combining an organometallic unit, i.e., metallocene with 4-amino-1,8-naphthalimide and a piperazine fragment have been studied, see Scheme 1. These molecules are designed according to the principles of photoinduced electron transfer (PET) systems. The groups are connected in an ‘electron donor–spacer1–fluorophore–spacer2–receptor’ format, where the electron donor is the metallocene. Metallocenes (M = Fe, Co, Ni), and specifically the M^2+^ cations, can act as electron donors responsive to M^3+^. The fluorophore is the 4-amino-1,8-naphthalimide and the terminal fragment is a piperazine unit which is easily protonated and acts as a proton receptor. Recently, Magri et al. have studied eight 4-amino-1,8-naphthalimide–ferrocene conjugates as potential multi-targeted anticancer agents [14].

The present calculated molecular systems have not been examined before. In the case of the Fe cation, the studied molecule is a truncated molecule of the three-input AND molecular logic gate, which has been studied experimentally by Magri [17] and theoretically by our group [18]. In the case of the Co cation and Ni cation, there are no experimental or theoretical studies on sensors or molecular logic gates that include cobaltocene or nickelocene groups. Thus, here we study the photophysical properties of molecular systems with potential interest as sensors. Furthermore, the effect of the substitution of the transition metal M (where M = Fe, Co, and Ni) in the 4-Amino-1,8-naphthalimide–metallocene conjugates regarding their oxidation state, and/or the protonation of the molecules is also studied.

## 2. Results and Discussion

### 2.1. Geometry

**Figure 1:**
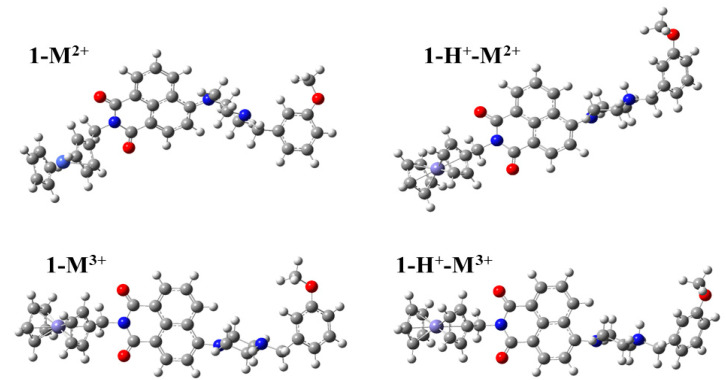
Calculated minimum structures at PBE0/6-31G(d,p). C atoms: grey balls, H atoms: white, O atoms: red; N atoms: blue; M (Fe, Co,Ni) atoms: violet.

Metallocene-4-amino-1,8-naphthalimide-piperazine molecules, **1-M^2+^**, where M = Fe, Co, and Ni, metallocenium derivatives, **1-M^3+^**, and the corresponding protonated derivatives, i.e., **1-M^2+^-H^+^** and **1-M^3+^-H^+^**, respectively, were studied via density functional theory (DFT) and time-dependent DFT (TD-DFT) using three functionals, i.e., PBE0, TPSSh and wB97XD in conjunction with the 6-31G(d,p) basis set, see Figure 1.

It was found that the three used functionals, i.e., PBE0, TPSSh and wB97XD, resulted in similar geometries, i.e., bond distances, angles, and dihedral angles, for the **1-M^2+^**, **1-M^3+^**, **1-M^2+^-H^+^,** and **1-M^3+^-H^+^** species, where M = Fe, Co, and Ni, as expected. Any differences in the geometries calculated by the three functionals, are expected only in the bond distances between the metal cation and the cyclopentadienyl ring, due to the application of different-type functionals, which differ in the inclusion of a dispersion model (wB97XD), or not (PBE0, TPSSh), and belong to different categories, i.e., hybrid-GGA (PBE0), hybrid meta-GGA(TPSSh), and range-separated hybrids(wB97XD). In general, we found that the use of wB97XD leads to an elongated M^2+/3+^…C_5_H_5_ bond distance, except for **1-Ni^3+^** and **1-Ni^3+^-H**^+^, see Table 1. However, whereas the discrepancies in these bond distances were up to ±0.1 Å, their average differences were up to ±0.01 Å, see Table 1. Overall, the M^2+/3+^…C_5_H_5_ bond distances range from 1.61 Å to 1.81 Å; the elongated values were observed for M = Ni, whereas iron and cobalt presented similar bond distances.

Furthermore, given that it was found that the geometry of the N atoms can lead to a shift in absorption spectra [18], the CNCC dihedral angles of all three N atoms were examined to check if their values are affected by the type of functional used. Their values are given in Table 1. It is interesting that the three N atoms of the twelve calculated structures have different geometries, which are similar in all three functionals for each structure. The N atom of pyridine has an almost planar geometry, i.e., the CNCC angle of pyridine (d_1_ value of Table 1) is ca. 178 degrees, whereas the N atom of the piperazine towards the phenyl group has an almost tetrahedral geometry that ranges from 124 to 127 degrees (d_3_ value of Table 1). The most important geometry is that of the N atom of piperazine towards the metallocene or metallocenium. The corresponding CNCC dihedral angle has intermediate value between planar and tetrahedral geometry that ranges from 140 to 153 degrees (d_2_ value of Table 1). **1-Co^2+^** and **1-Co^3+^** present the largest d_2_ values, up to 153 degrees, whereas the remaining ten species have d_2_ values that range from 140 to 144 degrees. This small increase in the d_2_ values of **1-Co^2+^** and **1-Co^3+^** leads to a significant red shift of the main peak of the absorption spectra, see below. It should be noted that similar behavior was found in our previous study on a 3-input AND molecular logic gate [18], where changes in the corresponding CNCC dihedral angle of about 10 degrees caused significant shifts of the main peak of the absorption spectra up to 100 nm.

Finally, in order to check the effect of the dispersion correction on geometries, the D3 version of Grimme’s dispersion correction was included in the geometry optimization of the **1-Fe^2+^** and **1-Co^3+^** using the PBE0-D3 and TPSSh-D3 functionals. These two molecules, **1-Fe^2+^** and **1-Co^3+^**, were selected because, in the first molecule, no differences among the geometries of the three functionals were observed, whereas, in the second one, the wB97XD functional predicts different d_2_ dihedral angles. d_2_ is an important dihedral angle, since small changes in its value affect the relative position of the main peak [18]. It should be noted that the dispersion correction is added in the calculated absolute energy, and this can affect the optimized geometry. It was found that the inclusion of D3 slightly decreases the PBE0 and TPPSSh M^2+/3+^…C_5_H_5_ bond distances, up to 0.01 Å, and it decreases the d_2_ dihedral angle by up to 4.7 degrees; the largest differences were observed for the **1-Co^3+^** molecule using the TPSSh-D3 functionals, whereas the smallest ones were observed for the **1-Fe^2+^** molecule using the PBE0-D3, which were only 0.001 Å and 1.0 degrees, respectively. To sum up, the inclusion of D3 dispersion correction only slightly affects the geometry and the largest effect is observed for the TPSSh-D3 functional, in comparison to TPSSh.

### 2.2. Energetics

The oxidation energy (OE) and the binding energy (BE) of the attached H^+^ in the complexes are given in Table 2. All the used functionals presented similar results. The differences in OE and BE using different functionals are less than 0.2 eV, except for the OE of **1-Co^2+^** and the BE of H^+^ in **1-Co^2+^-H^+^**, where the differences between TPSSh and the other two functionals is 0.3 eV. Note that the other two functionals predict the same values for these cases, i.e., the oxidation energies differ by 0.01 and the binding energies differ by 0.04 eV.

**Figure 2:**
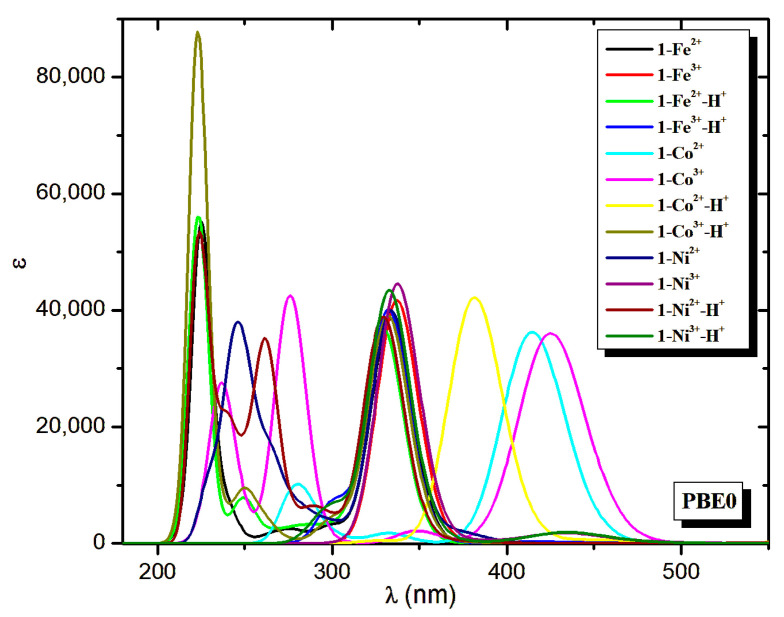
Absorption spectra of the calculated minima at PBE0/6-31G(d,p) in THF solvent.

**Figure 3:**
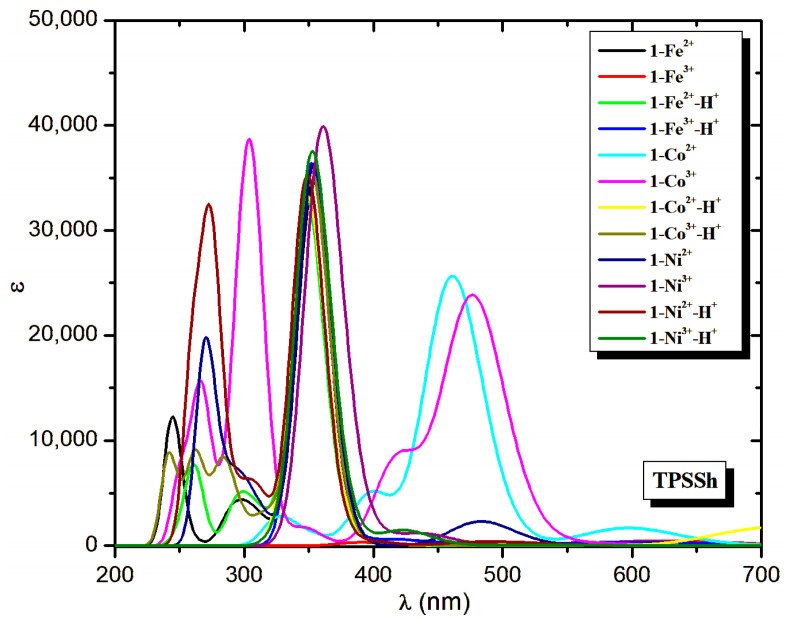
Absorption spectra of the calculated minima at TPSSh/6-31G(d,p) in THF solvent.

**Figure 4:**
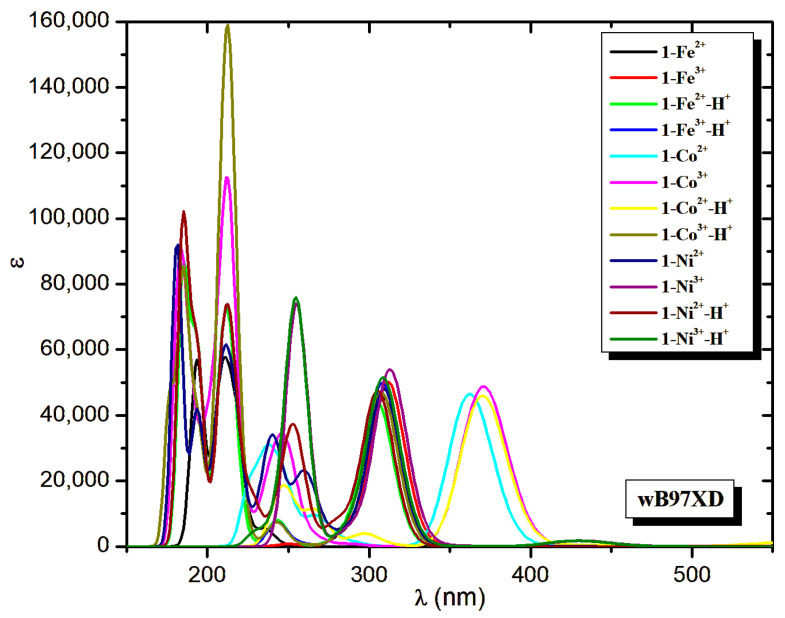
Absorption spectra of the calculated minima at wB97XD/6-31G(d,p) in THF solvent.

The Fe complexes present larger oxidation energies than the cobalt and nickel complexes, whereas, in the gas phase, the oxidation reaction of **M^2+^ → M^3+^** increase slightly from iron to nickel, i.e., the values are 30.651 (Fe^2+^), 33.50 (Co^2+^) and 35.187 (Ni^2+^) [41,42]. The corresponding oxidation energy values of all three functionals used are in excellent agreement with the experimental ones, and the best one is PBE0, see Table 2. Regarding the complexes, all functionals predict similar oxidation energies. We can consider that our best values are those obtained using the PBE0 functional, based on our study [18], where it was shown that PBE0 calculates the absorption spectra of azacrown ether derivatives of Fe complexes in excellent agreement with the experimental ones [18]. Thus, our best result for the oxidation energy of **1-Fe^2+^** is 5.30 eV, whereas, for the protonated complexes, it is increased to 5.45 eV. Note that the wB97XD values are up to 0.2 eV larger. The oxidation energies for cobalt and nickel complexes are smaller, at about 3.5 eV, see Table 2. The smallest oxidation energy is observed for the **1-Co^2+^-H^+^,** at 3.28 eV. It is of interest that the oxidation energy of the Fe^2+^ complex is about 17% of the free Fe^2+^ in the gas phase and, although the free Fe^2+^ has the smallest oxidation energy in the complexes, it has the largest compared to the other two metals. Finally, it should be mentioned that there are many intense UV absorption peaks in the area of 5.4 eV (230 nm), where Fe^2+^ is oxidized, and 3.2–3.5 eV (350–380 nm) where Co^2+^ and Ni^2+^ are oxidized, see Figure 2, Figure 3 and Figure 4 below, showing that the emission from these excited states can be antagonistic to their oxidation.

Finally, the binding energy of the attached proton ranges from 7.67 eV (**1-Co^2+^-H^+^**) to 8.27 eV (**1-Fe^2+^-H^+^**, **1-Ni^2+^-H^+^**). The wB97XD and TPSSh functionals predict the same values, or larger up to 0.2 eV, compared to PBE0. In the case of iron and nickel, the M^3+^ has a smaller BE, up to 0.2 eV, than the M^2+^ in all three functionals. For the cobalt complexes the opposite occurs only when using the PBE0 and wB97XD functionals, i.e., the Co^3+^ has the largest oxidation energy.

### 2.3. Absorption Spectra

The absorption spectra of the calculated minimum structures via the three functionals used are shown in Figure 2, Figure 3 and Figure 4. It was found that the spectra calculated using the three functionals are similar, however the TPSSh main peaks are red-shifted compared to the PBE0 ones by about 20 nm (0.2 eV), whereas the wB97XD main peaks are blue-shifted compared to the PBE0 ones by about 25 nm (0.3 eV) for the iron and nickel complexes and for **1-Co^3+^-H^+^**. In the cases of the **1-Co^3+^** and **1-Co^3+^,** both TPSSh and PBE0 present increased shifts, i.e., red shifts of 50 nm (TPSSh) and blue shifts of 50 nm (wB97XD). For the **1-Co^2+^-H^+^**, both TPSSh and wB97XD present blue shifts of 32 and 10 nm, respectively. Thus, except for **1-Co^2+^-H^+^**, the PBE0 functional predicts the main peaks having λ values that are between the values of the other two functionals and very close to the averages of the corresponding values of TPSSh and wB97XD. To sum up, we can conclude that PBE0, which has been proven to predict energetics well, and in excellent agreement with the experiment, see above and reference [18], is our best functional.

The main absorption peak for the iron and nickel complexes is at about 330 nm, i.e., it ranges from 329 nm (**1-Fe^2+^-H^+^** and **1-Ni^2+^-H^+^**) to 337 nm (**1-Fe^3+^** and **1-Ni^3+^**). It was observed that the main peak of the M^3+^ complexes is slightly red-shifted, i.e., by only 4 nm, compared to the M^2+^ complexes and the Fe and Ni present the same absorption spectra, see Table 3 and Figure 2 and Figure 4. The main peaks of **1-Co^3+^**, **1-Co^3+^** and **1-Co^2+^-H^+^** are in the visible area at 415 nm, 426 nm, and 381 nm, whereas the **1-Co^3+^-H^+^** is in the near-UV area at 332 nm. These differences in main peaks are a clue that the **1-Co^2+^** can be used as a sensor or as an MLG. Specifically, both the **1-Co^2+^** and **1-Co^3+^** can be used as a simple NOT MLG at their corresponding main peaks where the input is the protonation. This conclusion is supported by all three used functionals. Moreover, the **1-Co^2+^-H^+^** can also be used as a NOT MLG, where the input is the oxidation. Finally, the **1-Co^2+^** can be considered as a two-input NAND MLG, when the oxidation and the protonation are considered as inputs. The last conclusion is supported by both PBE0 and wB97XD data.

**Figure 5:**
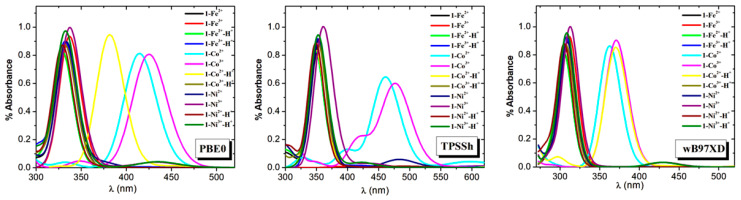
Percentage absorbance vs wavelength for the calculated minima in THF solvent.

However, it is interesting to analyze the differences obtained in the geometry and the corresponding absorption spectra for the three functionals. Although PBE0 and wB97XD calculate almost the same d_2_ dihedral angle for iron and nickel and the differences in d_2_ dihedral angles are less than 0.9 degrees, for **1-Co^2+^** and **1-Co^3+^**, the differences in d_2_ dihedral angles are about 5 degrees. It has been established that the d_2_ dihedral angle is crucial for the calculation of the spectra [18] and small differences, up to 10 degrees, can cause significant shifts in the main peak of the absorption spectra of up to 100 nm. Here, we found that the very small differences in geometries, that are common when different methodologies are used, including different functionals, can cause additional significant shifts in the measured major peaks of the absorption spectra, see Figure 5. Thus, for the **1-Co^2+^** and **1-Co^3+^** molecules, the ~5 degree decrease in the wB97XD d_2_ value (~146 degrees), compared to PBE0 and TPSSh d_2_ values (~151 degrees), results in a blue-shifted wB97XD main peak (~370), by about 50 nm compared to PBE0 (~425), and by about 100 nm compared to TPSSh (~477). On the contrary, in the molecules, where all three functionals have similar d_2_ values, the corresponding blue-shifted wB97XD main peak values are ~20 nm and ~50 nm, respectively. To sum up, the usually observed differences in the absorption spectra observed due to the different functionals used can be further increased when the functionals predict geometries with only small differences.

Furthermore, the D3 dispersion correction has been added to the geometry optimization of the **1-Fe^2+^** and **1-Co^3+^** using the PBE0 and TPSSh functionals to check the effect of the dispersion correction on the geometry and, consequently, on the absorption spectrum. It was found that the small differences in geometry between PBE0 and PBE0-D3 or TPSSh and TPSSh-D3 result only in a slight shift of the main peak, less than 3 nm, see Table 4. The largest shift was found for the **1-Co^3+^** molecule using the TPSSh and TPSSh-D3 functionals. So, the inclusion of the dispersion correction does not affect the absorption spectra.

Finally, the lowest S→S and S→T excitations, for the 1-M^2+^ and 1-M^2+^-H complexes, where M = Fe and Ni, are energetically degenerate for each complex. The protonation results in a red shift of about 45 nm^−1^ for this excitation. In the case of Fe, these excitations are at 444 nm and 473 nm (protonated complex), whereas the case of Ni corresponds to 784 nm and 834 nm (protonated complex). For the Co^3+^ complexes, the S→T excitations are at about 600 nm, whereas, for the Co^2+^ complexes, the lowest-in-energy D→D excitation is at 620 nm, see Table 3.

### 2.4. Molecular Orbitals

**Figure 6:**
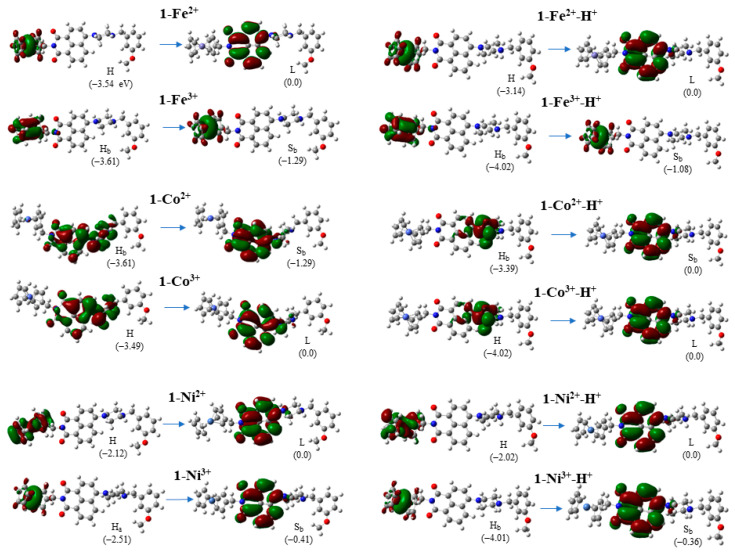
H, L, or S molecular orbitals of the calculated minima structures involved in the first absorption peak in the visible area at PBE0/6-31G(d,p) in THF solvent. Relative energies of orbitals in eV are included with respect to the L orbital. C atoms: grey balls, H atoms: white, O atoms: red; N atoms: blue, M (Fe, Co,Ni) atoms: violet.

The lowest-in-energy excitations, H→L or H→S (for doublets), are given in Figure 6. For the iron and nickel complexes, these excitations are electron transfer from metallocene or metallocenium group to 4-amino-1,8-naphthalimide and this is the reason for their very small, or even zero, oscillator strength. In the case of cobalt, the corresponding lowest-in-energy excitations, H→L or H→S (for doublets), are not charge transfer excitations, the electron density is localized at 4-amino-1,8-naphthalimide and piperazine fragments, or in the case of the protonated complexes, there is a partial charge transfer from piperazine to 4-amino-1,8-naphthalimide.

**Figure 7:**
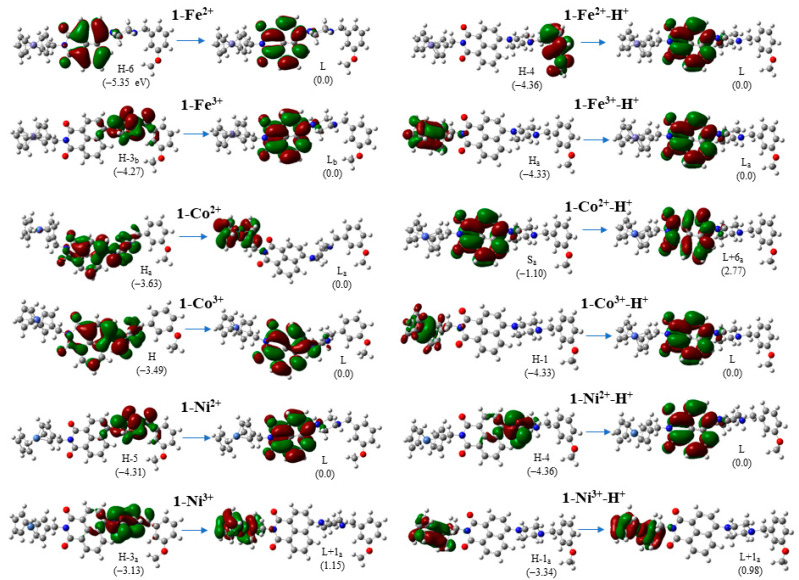
Frontier molecular orbitals of the calculated minima structures involved in the main absorption peaks in visible and near-UV areas (Table 4) at PBE0/6-31G(d,p) in THF solvent. Relative energies of orbitals in eV are included with respect to the L orbital. C atoms: grey balls, H atoms: white, O atoms: red; N atoms: blue, M (Fe, Co,Ni) atoms: violet.

Regarding the main absorption peaks in visible and near-UV areas (Table 4), it is interesting that most of them are charge transfer (CT) excitations, see Figure 7. The charge is transferred, in most cases, from metallocene/metallocenium or piperazine to fluorophore, i.e., 4-amino-1,8-naphthalimide. The charge is transferred in metallocene/metallocenium only in the case of **1-Co^2+^** and **1-Ni^3+^**, whereas, in the case of **1-Ni^3+^-H^+^,** that charge is maintained at nickelocenium. Finally, it should be noted that intense CT excitations, such as in the case of the present molecules, are not very common.

## 3. Computational Details

All calculations were carried out using the PBE0 [43,44], TPSSh [45], and wB97XD [46] functionals in conjunction with the 6-31G(d,p) [47] basis set in THF solvent employing the polarizable continuum model (PCM) [48,49]. The THF solvent was employed because it is a versatile solvent, with low viscosity and low dielectric constant, ε = 7.4257. All calculated molecular systems are soluble in THF solvent. Our previous calculations on derivatives of ferrocene species, including a crown ether receptor of the Na^+^ cation, show that the PBE0/6-31G(d,p) [18] is a very good choice for these systems; the computational data, including absorption and emission spectra, were in excellent agreement with the experimental spectra [17]. Here, three different hybrid types of functionals were used for comparison reasons. PBE0, which is a hybrid GGA functional. The TPSSh functional, which is a hybrid meta-GGA one, and it is regarded as a very good choice for the first-row transition metals [28], and the wB97XD functional, which is a range-separated hybrid functional, using the Grimme’s D2 dispersion model, and includes long range corrections [46]. Finally, the D3 version of Grimme’s dispersion correction [50] was added to PBE0 and TPSSh functionals, i.e., PBE0-D3 and TPSSh-D3, to check if the inclusion of the dispersion correction affected the geometry, and consequently the absorption spectra, of the calculated molecules.

At first, conformational analyses were carried out, where all species were fully energetically optimized in the ground state to locate a global minimum structure for each species. The energy convergence criteria for the geometry optimization were set to 1 × 10^−8^ hartree. The absorption spectra of the studied structures were calculated via the DFT methodology in THF solvent. In all cases, the absorption spectra of the studied systems were calculated including the 50 lowest-in-energy excited singlet-spin electronic states and the 50 lowest-in-energy excited triplet-spin electronic states. The UV-Vis peak, half width at half height, was 0.2 eV. Additionally, the application of the linear response correction (cLR) approach for the main absorption peaks was checked and it was found that it resulted in differences smaller than 3 nm, see also ref. [18]. All calculations were carried out employing the Gaussian16 code [51].

## 4. Conclusions

In the present paper, we studied the photophysical properties of molecular systems with potential interest as sensors, i.e., metallocene-4-amino-1,8-naphthalimide-piperazine molecules, **1-M^2+^**, metallocenium derivatives, **1-M^3+^**, and the corresponding protonated derivatives, i.e., **1-M^2+^-H^+^** and **1-M^3+^-H^+^**, where M = Fe, Co, and Ni, via DFT and TD-DFT methodologies. The effect of substitution of the transition metal M on their oxidation state, and/or the protonation of the molecules, was calculated using three different functionals, i.e., PBE0, TPSSh, and wB97XD.

These molecules generate research interest for potential applications for the following reasons: they combine an organometallic unit, i.e., metallocene, which can easily be oxidized; they contain an attractive electrondeficient moiety, i.e., the 1,8-naphthalimide group, which has been widely studied as its derivatives can be easily synthesized with low cost [26,27] and have various technological applications, such as in OPVs, OLEDs, bioimaging, pH and metal sensors, laser and fluorescent dyes, etc.; and they possess a terminal fragment, i.e., a piperazine unit, which is easily protonated and acts as a proton receptor.

Regarding the geometry, all three functionals predict similar geometry for all calculated molecules. Specifically, concerning the geometry of the N atoms, the N atom of the pyridine has a planar geometry, whereas the N atom of the piperazine towards the phenylene group has a tetrahedral geometry. The N atom of piperazine towards metal complexes has intermediate values between planar and tetrahedral geometry that range from 140 to 153 degrees. **1-Co^2+^** and **1-Co^3+^** present the largest d_2_ values, up to 153 degrees, whereas the remaining ten species have d_2_ values that range from 140 to 144 degrees. This small increase in the d_2_ values of **1-Co^2+^** and **1-Co^3+^** leads to a significant red shift of the main peak of the absorption spectra.

All three functionals predict similar spectra. In the UV area, there are small differences in the calculated spectra, whereas, for the main peak in visible or near-UV areas, differences of up to 100 nm are observed. Our best results are obtained with the PBE0 functional. PBE0, which has predicted absorption and emission spectra that are in excellent agreement with experimental ones for similar systems [17,18], here predicts main peaks having average λ values with respect to the other two functionals, i.e., the TPSSh functional predicts main peaks that are red-shifted by up to 50 nm, whereas the wB97XD functional predicts main peaks that are blue-shifted by up to 50 nm with respect to the PBE0 functional.

We found that very small differences in geometries (CNCC dihedral angle of piperazine) can cause significant shifts in the absorption spectra of up to 100 nm. Furthermore, the common differences in spectra due to the use of different functionals (20 to 50 nm) can be further increased, they are doubled, when the functionals predict geometries with only small differences in geometry.

The lowest-in-energy excitations, H→L or H→S (for doublets), are electron transfers from the metallocene or metallocenium group to 4-amino-1,8-naphthalimide with a very small, or even zero, oscillator strength. In most molecules, the main absorption peaks in visible and near-UV areas correspond mainly to charge transfer (CT) excitations, where the charge is transferred from metallocene/metallocenium or piperazine to fluorophore.

The Fe complexes present larger oxidation energies than cobalt or nickel complexes. All functionals predict similar oxidation energies. Our best result for the oxidation energy of **1-Fe^2+^** is 5.30 eV, whereas, for the protonated complexes, it is higher, at 5.45 eV. The oxidation energies for cobalt and nickel complexes are smaller, at about 3.5 eV. Finally, it should be mentioned that there are many intense UV absorption peaks with excitation energies similar to their oxidation energy, showing that the emission from these excited states can be antagonistic to their oxidation. The binding energy of the attached proton ranges from 7.67 eV (**1-Co^2+^-H^+^**) to 8.27 eV (**1-Fe^2+^-H^+^**, **1-Ni^2+^-H^+^**). Thus, the replacement of Fe with Co or Ni in the present calculated metallocene-naphthalimide derivatives significantly lowers the oxidation energy, i.e., it facilitates the oxidation. The replacement of Fe with Ni results in similar absorption spectra and protonation energies.

Additional important outcomes of the present computational study are: (1) the inclusion of dispersion corrections does not affect the calculation of the geometry and consequently the absorption spectra of these systems; (2) for certain applications, where there is a need for a redox molecular system including metallocene, in which the oxidation energies should be lowered significantly, to about 40%, this can happen via the replacement of the iron with cobalt or nickel; and (3) the present system, using cobalt as the transition metal, has the potential to be used as a sensor using the absorption spectra as an output.

Finally, it should be noted that the present calculated systems have not been theoretically or experimentally investigated before. The present study adds data regarding the photophysical properties of calculated molecular systems in order to provide experimentalists with information about these systems. Note that, only in the case of iron have derivatives been synthesized, and it was found that they have interesting properties with regard to their UV-vis spectra. Furthermore, the present study provides important information regarding the effect of geometry and functionals on absorption spectra.

## Figures and Tables

**Table 1:** Selected distances (Å) and dihedral angles (degrees) of the calculated structures at PBE0, TPSSh, and wB97XD/6-31G(d,p) in THF solvent.

Molecule	R ^a^	d_1_^b^	d_2_^b^	d_3_^b^		R ^a^	d_1_^b^	d_2_^b^	d_3_^b^		R ^a^	d_1_^b^	d_2_^b^	d_3_^b^
	PBE0					TPSSh					wB97XD			
**1-Fe^2+^**	1.623	178.2	141.8	124.9		1.613	178.9	140.1	124.3		1.632	176.1	141.4	125.4
^c^	1.622	175.9	140.8	125.9		1.605	173.8	138.8	125.3					
**1-Fe^3+^**	1.642	177.9	142.1	124.8		1.637	178.6	140.4	124.2		1.645	175.8	141.8	125.4
**1-Fe^2+^-H^+^**	1.621	177.9	143.2	126.7		1.613	178.5	141.6	126.6		1.631	175.7	142.9	126.7
**1-Fe^3+^-H^+^**	1.642	177.9	143.7	126.7		1.681	178.2	142.3	126.7		1.645	175.0	143.4	126.7
**1-Co^2+^**	1.695	178.7	150.0	125.0		1.664	179.2	150.2	124.4		1.717	177.3	145.6	124.9
**1-Co^3+^**	1.610	178.9	152.4	125.1		1.606	179.6	152.9	124.6		1.616	176.8	147.0	125.1
^c^	1.607	176.6	149.7	125.4		1.596	173.7	148.2	124.6					
**1-Co^2+^-H^+^**	1.610	178.2	142.1	126.7		1.661	177.9	141.6	126.6		1.615	176.5	141.4	126.5
**1-Co^3+^-H^+^**	1.610	178.1	143.7	126.7		1.606	178.8	142.1	126.6		1.616	175.6	143.4	126.7
**1-Ni^2+^**	1.756	178.0	141.9	124.8		1.755	178.2	140.2	124.2		1.781	176.5	141.3	125.4
**1-Ni^3+^**	1.720	178.2	142.1	126.7		1.692	179.1	140.5	124.2		1.602	176.8	141.8	125.3
**1-Ni^2+^-H^+^**	1.658	179.2	143.1	126.5		1.809	180.0	141.6	126.4		1.796	177.1	142.7	126.5
**1-Ni^3+^-H^+^**	1.694	179.0	143.8	126.8		1.679	178.5	142.2	126.4		1.604	176.5	143.1	126.6
Average/Fe ^d^	1.632	178.0	142.7	125.8		1.636	178.6	141.1	125.4		1.638	175.7	142.4	126.0
Average/Co ^d^	1.631	178.5	147.0	125.9		1.634	178.9	146.7	125.6		1.641	176.5	144.3	125.8
Average/Ni ^d^	1.707	178.6	142.7	126.2		1.734	178.9	141.1	125.3		1.696	176.7	142.2	125.9
Average/M ^d^	1.656	178.3	144.1	125.9		1.668	178.8	143.0	125.4		1.658	176.3	143.0	125.9

^a^ R distance of the metallocene from the center of each cyclopentadienyl ring in the metallocene unit (average values of R_1_ and R_2_, see Scheme 1). ^b^ Dihedral CNCC angles of pyridine (d_1_) and piperazine (d_2_: towards ferrocene and d_3_: towards phenylene group), i.e., dihedral angles of the N atom with its nearby connected C atoms, see Scheme 1. ^c^ Including the D3 version of Grimme’s dispersion correction, PBE0-D3 and TPSSh-D3. ^d^ Average values for Fe, Co, Ni and M, where M represents all three metals.

**Table 2:** Oxidation Energy M^2+^ → M^3+^ (OE in eV) in the Calculated Complexes, in eV, and Binding energy of H^+^ (BE in eV) in THF solvent, see Scheme 1.

	PBE0	TPSSh	wB97XD	Expt		PBE0	TPSSh	wB97XD
		OE				BE		
**1-Fe^2+^ → 1-Fe^3+^**	5.30	5.38	5.45		**1-Fe^2+^-H^+^**	8.27	8.36	8.38
**1-Fe^2+^-H^+^ → 1-Fe^3+^-H^+^**	5.45	5.62	5.66		**1-Fe^3+^-H^+^**	8.12	8.12	8.17
**1-Co^2+^ → 1-Co^3+^**	3.49	3.17	3.48		**1-Co^2+^-H^+^**	7.67	8.12	7.71
**1-Co^2+^-H^+^ → 1-Co^3+^-H^+^**	3.28	3.35	3.14		**1-Co^3+^-H^+^**	7.87	7.94	8.05
**1-Ni^2+^ → 1-Ni^3+^**	3.55	3.38	3.57		**1-Ni^2+^-H^+^**	8.27	8.37	8.41
**1-Ni^2+^-H^+^ → 1-Ni^3+^-H^+^**	3.69	3.53	3.74		**1-Ni^3+^-H^+^**	8.13	8.22	8.24
**Fe^2+^ → Fe^3+^**	30.97	31.13	31.04	30.651 ^a^				
**Co^2+^ → Co^3+^**	33.35	33.40	33.82	33.5 ^a^				
**Ni^2+^ → Ni^3+^**	35.20	35.04	33.68	35.187 ^b^				

^a^ Reference [41]. ^b^ Reference [42].

**Table 3:** Absorption maxima and charge transfer peaks, λ (nm), energy differences, ΔΕ (eV), of S_0_→S_x_, S_0_→T_1_ and D→D, f-values, and the corresponding main excitations of the absorption spectra of the **1-M^2+^**, **1-M^3+^**, **1-M^2+^-H^+^**, and **1-M^3+^-H^+^** species at the PBE0/6-31G(d,p) level of theory.

Molecule	Peaks	λ	ΔΕ	f	Main Excitation
**1-Fe^2+^**	*S→S*	443.7	2.795	0.0009	H → L
	*S→T*	443.5	2.796	0.0000	H → L
	*S→S*	333.5	3.718	0.4260	H-6 → L
**1-Fe^3+^**	*D→D*	597.4	2.076	0.0000	H_β_ → S_β_
	*D→D*	382.7	3.240	0.0000	H_β_ → L_β_
	*D→D*	377.9	3.281	0.0001	H_α_ → L_α_
	*D* *→* *D*	337.2	3.677	0.4444	H-3_β_ → L_β_
**1-Fe^2+^-H^+^**	*S→S*	475.7	2.607	0.0000	H → L
	*S→T*	473.4	2.619	0.0000	H → L
	*S→S*	329.2	3.766	0.4069	H-4 → L
**1-Fe^3+^-H^+^**	*D→D*	455.0	2.725	0.0000	H_β_ → S_β_
	*D→D*	339.2	3.656	0.0019	S_α_ → L_α_
	*D→D*	339.1	3.657	0.0213	H_β_ → L_β_
	*D* *→* *D*	331.8	3.736	0.3888	H_α_ → L_α_
**1-Co^2+^**	*D→D*	619.6	2.001	0.0000	H_β_ → S_β_
	*D* *→* *D*	414.9	2.988	0.3969	H_α_ → L_α_
	*D→D*	334.2	3.710	0.0000	H_β_ → L_β_
**1-Co^3+^**	*S→* *T*	630.5	1.967	0.0000	H → L
	*S→S*	425.8	2.912	0.3871	H → L
**1-Co^2+^-H^+^**	*D→D*	436.7	2.839	0.0001	H_β_ → S_β_
	*D* *→* *D*	380.5	3.259	0.2684	S_α_ → L+6_α_
**1-Co^3+^-H^+^**	*S→* *T*	605.7	2.047	0.0000	H → L
	*S→S*	339.3	3.654	0.0171	H → L
	*S→S*	331.7	3.738	0.4204	H-1 → L
**1-Ni^2+^**	*S→T*	783.6	1.582	0.0000	H → L
	*S→S*	781.0	1.588	0.0006	H → L
	*S→S*	333.7	3.715	0.4337	H-5 → L
**1-Ni^3+^**	*D→D*	565.5	2.193	0.0001	S_α_ → L_α_
	*D→D*	442.3	2.803	0.0000	H_β_ → S_β_
	*D→D*	385.2	3.219	0.0004	H_β_ → L_β_
	*D* *→* *D*	337.4	3.675	0.3463	H-3_α/β_ → L+1_α/β_
**1-Ni^2+^-H^+^**	*S→* *T*	834.0	1.487	0.0000	H → L
	*S→S*	833.2	1.488	0.0001	H → L
	*S→S*	329.5	3.763	0.4245	H-4 → L
**1-Ni^3+^-H^+^**	*D→D*	361.5	3.430	0.0001	H_β_ → S_β_
	*D→D*	339.9	3.648	0.0184	H_β_ → L_β_
	*D* *→* *D*	332.0	3.735	0.2374	H-1_α_ → L+1_α_

**Table 4:** Main absorption peaks, λ (nm), energy differences, ΔΕ (eV), and f-values of the **1-M^2+^**, **1-M^3+^**, **1-M^2+^-H^+^**, and **1-M^3+^-H^+^** species at the PBE0, TPSSh, and wB97XD/6-31G(d,p) level of theory.

Molecule	λ	ΔΕ	f		λ	ΔΕ	f		λ	ΔΕ	f
	PBE0				TPSSh				wB97XD		
**1-Fe^2+^**	333.5	3.718	0.426		353.5	3.508	0.234		308.9	4.013	0.503
^a^	333.5	3.717	0.425		353.9	3.503	0.274				
**1-Fe^3+^**	337.2	3.677	0.444		357.4	3.469	0.250		311.9	3.975	0.551
**1-Fe^2+^-H^+^**	329.2	3.766	0.407		348.5	3.557	0.365		305.3	4.062	0.484
**1-Fe^3+^-H^+^**	331.8	3.736	0.389		352.1	3.521	0.278		308.4	4.020	0.356
**1-Co^2+^**	414.9	2.988	0.397		461.2	2.689	0.277		362.5	3.421	0.517
**1-Co^3+^**	425.8	2.912	0.387		477.2	2.598	0.262		370.8	3.344	0.541
^a^	426.0	2.910	0.309		480.7	2.579	0.240				
**1-Co^2+^-H^+^**	380.5	3.259	0.268		348.3	3.560	0.339		370.3	3.349	0.510
**1-Co^3+^-H^+^**	331.7	3.738	0.420		352.4	3.518	0.336		307.8	4.028	0.516
**1-Ni^2+^**	333.7	3.715	0.434		353.7	3.506	0.187		309.2	4.010	0.533
**1-Ni^3+^**	337.4	3.675	0.346		359.9	3.445	0.124		311.6	3.979	0.370
**1-Ni^2+^-H^+^**	329.5	3.763	0.425		348.6	3.556	0.329		305.4	4.059	0.523
**1-Ni^3+^-H^+^**	332.0	3.735	0.237		352.2	3.520	0.279		308.0	4.026	0.501

^a^ Including the D3 version of Grimme’s dispersion correction, PBE0-D3 and TPSSh-D3.

## Data Availability

Data are provided in Supporting Information.

## Supplementary Materials

The following supporting information can be downloaded at https://www.mdpi.com/article/10.3390/molecules28083565/s1: Computational Details and Geometries of the calculated minimum structures.

## Sample Availability

Samples of the compounds are available from the authors.
