# Supplementary material for: Metallocene-Naphthalimide Derivatives: The Effect of Geometry, DFT Methodology, and Transition Metals on Absorption Spectra
Source: Molecules. 2023 Apr 19;28(8):3565. doi: 10.3390/molecules28083565 (PMC10146125; doi:10.3390/molecules28083565)
Supplement: Supplementary file 1 [file molecules-28-03565-s001.zip › molecules-2300880-supplementary.pdf]

Supporting Information

Metallocene-naphthalimide derivatives: The effect of geometry, DFT methodology, and transition metals on absorption spectra

Christina Eleftheria Tzeliou[a] and Demeter Tzeli[a,b*]

[a] Laboratory of Physical Chemistry, Department of Chemistry, National and Kapodistrian University of Athens, Panepistimiopolis Zografou, Athens 157 84, Greece

[b] Theoretical and Physical Chemistry Institute, National Hellenic Research Foundation, 48 Vassileos Constantinou Ave., Athens 116 35, Greece

Computational Details:

Methodology: PBE0/6-31G(d,p) TPSSh/6-31G(d,p) and wB97XD/6-31G(d,p)
The basis set contains 816 basis functions, i.e., 816 molecular orbitals.
157 molecular orbitals are double occupied and 659 molecular orbitals are unoccupied.

At first, conformational analyses were carried out, where all species were fully energetically optimized in the ground. The energy convergence criteria for the geometry optimization are set to 1×10^{-8} hartree.

Threshold Convergence criteria: Maximum Force < 0.000450; RMS Force < 0.000300;
Maximum Displacement < 0.001800; RMS Displacement < 0.001200

Geometry Optimization examples

```
# opt pbe1pbe/6-31g(d,p) scrf=(solvent=thf)
# opt tpssh/6-31g(d,p) scrf=(solvent=thf)
# opt wb97xd/6-31g(d,p) scrf=(solvent=thf)
# opt pbe1pbe/6-31g(d,p) scrf=(solvent=thf) EmpiricalDispersion=GD3
# opt tpssh/6-31g(d,p) scrf=(solvent=thf) EmpiricalDispersion=GD3 IOp(3/174=1000000) IOp(3/175=1000000)
IOp(3/176=1000000)
```

The absorption spectra of the studied structures were calculated via the DFT methodology in THF solvent. In all cases, the absorption spectra of the studied systems were calculated including the 50 lowest in energy excited singlet-spin electronic states and the 50 lowest in energy excited triplet-spin electronic states. The UV-Vis peak half-width at half height is 0.2 eV.

Absorption spectrum calculation examples

```
# td=(singlets,nstates=50,root=1) pbe1pbe/6-31g(d,p) scrf=(solvent=thf)
# td=(singlets,nstates=50,root=1) pbe1pbe/6-31g(d,p) scrf=(solvent=thf) guess=save EmpiricalDispersion=GD3
# td=(singlets,nstates=50,root=1) tpssh/6-31g(d,p) scrf=(solvent=thf) guess=save EmpiricalDispersion=GD3
IOp(3/174=1000000) IOp(3/175=1000000) IOp(3/176=1000000)
```

Geometries obtained at PBE0/6-31G(d,p)

❖ **1-Fe^{2+}**

Center Number	Atomic Number	Atomic Type	Coordinates (Angstroms) X	Y	Z
1	6	0	8.401527	-0.840209	0.262020
2	6	0	9.487885	-1.445114	-0.364650
3	6	0	8.302135	0.555708	0.286059
4	6	0	10.477559	-0.657104	-0.955352
5	1	0	9.562027	-2.528957	-0.392896
6	6	0	9.289572	1.337249	-0.312554
7	1	0	7.436061	0.998819	0.764681
8	6	0	10.386198	0.725652	-0.934148
9	1	0	11.325315	-1.128886	-1.444274
10	8	0	9.276431	2.694166	-0.343626
11	6	0	8.188296	3.355586	0.269127
12	1	0	7.235335	3.086202	-0.202239
13	1	0	8.132505	3.132603	1.341451
14	1	0	8.366666	4.422759	0.133337
15	6	0	7.353638	-1.675089	0.961019
16	1	0	7.563029	-1.671339	2.039306
17	1	0	7.447591	-2.727239	0.631396
18	6	0	5.542838	-1.327038	-0.590193
19	6	0	5.065964	-1.796811	1.699080
20	6	0	4.176927	-0.685251	-0.766579
21	1	0	5.479645	-2.396702	-0.868821
22	1	0	6.260079	-0.845474	-1.262388
23	6	0	3.692403	-1.159264	1.565056
24	1	0	4.978065	-2.884132	1.510946
25	1	0	5.428395	-1.661846	2.724393
26	1	0	3.810483	-0.873098	-1.780308
27	1	0	4.283959	0.406904	-0.645564
28	1	0	2.979750	-1.681034	2.210889
29	1	0	3.758021	-0.113668	1.912656
30	7	0	6.003752	-1.167646	0.781854
31	7	0	3.245599	-1.275641	0.184424
32	6	0	1.856806	-1.113403	-0.071735
33	6	0	1.165742	0.135047	0.050091
34	6	0	1.151952	-2.238283	-0.459003
35	6	0	1.799269	1.342660	0.435525
36	6	0	-0.231701	0.174174	-0.230803
37	6	0	-0.223108	-2.190433	-0.733803
38	1	0	1.696378	-3.172901	-0.544393
39	6	0	1.090504	2.519101	0.540580
40	1	0	2.860817	1.339957	0.650903
41	6	0	-0.939407	1.393567	-0.115789
42	6	0	-0.912509	-1.001077	-0.622438

43	1	0	-0.762409	-3.082607	-1.035058
44	6	0	-0.286305	2.547857	0.265807
45	1	0	1.597851	3.431074	0.838119
46	1	0	-0.856047	3.467652	0.346938
47	6	0	-2.358567	-0.971308	-0.913441
48	6	0	-2.387654	1.448617	-0.400920
49	8	0	-2.984197	-1.967461	-1.247843
50	8	0	-3.037690	2.480042	-0.305300
51	7	0	-2.999017	0.261113	-0.796931
52	6	0	-4.440063	0.304003	-1.078443
53	1	0	-4.634894	-0.448650	-1.842430
54	1	0	-4.654612	1.295020	-1.478638
55	6	0	-5.264049	0.039637	0.142332
56	6	0	-5.734345	1.017598	1.071237
57	6	0	-5.712697	-1.238040	0.596973
58	1	0	-5.565783	2.083406	0.992316
59	6	0	-6.478141	0.348202	2.082502
60	1	0	-5.525060	-2.178629	0.096345
61	6	0	-6.464700	-1.046360	1.789322
62	1	0	-6.989467	0.818156	2.912508
63	1	0	-6.963724	-1.820215	2.357952
64	6	0	-8.836747	-1.021212	-0.547049
65	6	0	-8.083934	-0.371644	-1.567072
66	1	0	-7.579636	-0.857643	-2.392147
67	6	0	-8.085065	1.026549	-1.291882
68	1	0	-7.581789	1.789666	-1.871239
69	6	0	-9.303041	-0.025334	0.357955
70	1	0	-9.885612	-0.201940	1.252677
71	6	0	-8.838491	1.239987	-0.101925
72	1	0	-9.005510	2.193237	0.382260
73	1	0	-9.002378	-2.087086	-0.460342
74	26	0	-7.277670	-0.003233	0.253973
75	1	0	11.143524	1.352568	-1.394353

❖ **1-Fe^{3+}**

Center Number	Atomic Number	Atomic Type	Coordinates (Angstroms) X	Y	Z
1	6	0	8.393047	-0.844173	0.224111
2	6	0	9.361501	-1.451352	-0.566986
3	6	0	8.400951	0.551250	0.331728
4	6	0	10.337664	-0.686778	-1.202499
5	1	0	9.374652	-2.533503	-0.673271
6	6	0	9.341265	1.325334	-0.341350
7	1	0	7.616951	1.022890	0.913891
8	6	0	10.363869	0.709651	-1.112682
9	1	0	11.119253	-1.166369	-1.784531
10	8	0	9.329729	2.687159	-0.325788

11	6	0	8.342970	3.335737	0.450068
12	1	0	7.332032	3.098202	0.097399
13	1	0	8.423279	3.070567	1.510997
14	1	0	8.521771	4.405050	0.332441
15	6	0	7.366219	-1.651971	0.978071
16	1	0	7.591109	-1.616993	2.052726
17	1	0	7.448016	-2.713989	0.677342
18	6	0	5.535483	-1.319679	-0.555084
19	6	0	5.085862	-1.773635	1.741075
20	6	0	4.166237	-0.682967	-0.722905
21	1	0	5.472626	-2.392130	-0.824167
22	1	0	6.244431	-0.842786	-1.239174
23	6	0	3.706419	-1.146191	1.617696
24	1	0	5.002520	-2.863105	1.561766
25	1	0	5.457840	-1.630002	2.761799
26	1	0	3.790467	-0.878076	-1.731836
27	1	0	4.272111	0.409587	-0.609246
28	1	0	3.004063	-1.669784	2.273247
29	1	0	3.765691	-0.097611	1.957474
30	7	0	6.010804	-1.146422	0.809733
31	7	0	3.246632	-1.272347	0.241322
32	6	0	1.855122	-1.121299	-0.000511
33	6	0	1.164188	0.131101	0.078763
34	6	0	1.146323	-2.261991	-0.331941
35	6	0	1.800199	1.348543	0.427013
36	6	0	-0.233528	0.162801	-0.201332
37	6	0	-0.229672	-2.224114	-0.599237
38	1	0	1.689235	-3.199698	-0.384753
39	6	0	1.095929	2.530827	0.492575
40	1	0	2.860325	1.349125	0.648598
41	6	0	-0.935845	1.389500	-0.130391
42	6	0	-0.916067	-1.028277	-0.541211
43	1	0	-0.769617	-3.128800	-0.858884
44	6	0	-0.279284	2.554903	0.212758
45	1	0	1.605535	3.449754	0.762733
46	1	0	-0.844004	3.480084	0.261036
47	6	0	-2.357961	-1.007668	-0.833826
48	6	0	-2.379758	1.439527	-0.419007
49	8	0	-3.002425	-2.010115	-1.108791
50	8	0	-3.042047	2.465264	-0.350293
51	7	0	-2.986606	0.238709	-0.791703
52	6	0	-4.408225	0.282531	-1.125404
53	1	0	-4.595562	-0.499659	-1.860435
54	1	0	-4.611853	1.260200	-1.561201
55	6	0	-5.247061	0.066558	0.099202
56	6	0	-5.724776	1.080585	0.987057
57	6	0	-5.690933	-1.193650	0.608847
58	1	0	-5.584738	2.144508	0.850415
59	6	0	-6.459767	0.450277	2.031079
60	1	0	-5.520682	-2.151549	0.135928

61	6	0	-6.438801	-0.956600	1.797165
62	1	0	-6.990901	0.953963	2.827565
63	1	0	-6.951069	-1.706067	2.385445
64	6	0	-8.868720	-1.050514	-0.507155
65	6	0	-8.148769	-0.437158	-1.573709
66	1	0	-7.639846	-0.954461	-2.376003
67	6	0	-8.158382	0.972383	-1.358481
68	1	0	-7.658414	1.712313	-1.969064
69	6	0	-9.323955	-0.020180	0.366485
70	1	0	-9.857412	-0.165885	1.296143
71	6	0	-8.884354	1.229875	-0.158990
72	1	0	-9.027102	2.198276	0.301250
73	1	0	-8.997746	-2.113910	-0.357142
74	26	0	-7.288339	0.015721	0.221517
75	1	0	11.111650	1.263954	-1.640385

❖ **$1-H^{+}-Fe^{2+}$**

Center Number	Atomic Number	Atomic Type	Coordinates (Angstroms) X	Y	Z
1	6	0	8.406971	-0.920862	0.145359
2	6	0	8.761359	-1.616973	-1.009539
3	6	0	9.060599	0.284302	0.441674
4	6	0	9.761263	-1.123881	-1.832197
5	1	0	8.297910	-2.571050	-1.247932
6	6	0	10.040471	0.800859	-0.400923
7	1	0	8.805824	0.828463	1.347342
8	6	0	10.429883	0.077788	-1.565236
9	1	0	10.079017	-1.686522	-2.705736
10	8	0	10.647884	1.985491	-0.169445
11	6	0	10.287414	2.711502	0.990334
12	1	0	9.223028	2.975962	0.976970
13	1	0	10.521527	2.148958	1.901459
14	1	0	10.886331	3.621024	0.961751
15	6	0	7.377811	-1.454383	1.075473
16	1	0	7.614659	-1.252663	2.124401
17	1	0	7.218265	-2.528457	0.950344
18	6	0	5.434508	-1.187811	-0.493764
19	6	0	5.044138	-1.090888	1.948672
20	6	0	4.095773	-0.499271	-0.699198
21	1	0	5.315955	-2.274054	-0.477159
22	1	0	6.170534	-0.907251	-1.250778
23	6	0	3.711858	-0.410553	1.676869
24	1	0	4.929539	-2.176716	1.988777
25	1	0	5.499123	-0.734866	2.876249
26	1	0	3.673123	-0.856857	-1.641650
27	1	0	4.257438	0.589602	-0.809594
28	1	0	3.012492	-0.705900	2.463470

29	1	0	3.842019	0.685461	1.754215
30	7	0	6.013800	-0.807304	0.836960
31	7	0	3.210290	-0.843099	0.392023
32	6	0	1.799243	-0.824354	0.164303
33	6	0	1.059001	0.385666	0.006237
34	6	0	1.156313	-2.043247	0.100313
35	6	0	1.639545	1.676125	0.061807
36	6	0	-0.344825	0.291585	-0.222283
37	6	0	-0.228052	-2.126949	-0.121032
38	1	0	1.743845	-2.946710	0.224837
39	6	0	0.876360	2.810868	-0.093485
40	1	0	2.706533	1.773948	0.229864
41	6	0	-1.107163	1.473084	-0.382117
42	6	0	-0.968587	-0.976567	-0.283284
43	1	0	-0.729766	-3.087879	-0.171225
44	6	0	-0.507559	2.713153	-0.315310
45	1	0	1.342469	3.789479	-0.046545
46	1	0	-1.120908	3.599465	-0.441424
47	6	0	-2.420580	-1.087112	-0.527396
48	6	0	-2.559725	1.395979	-0.630036
49	8	0	-3.022458	-2.145874	-0.551349
50	8	0	-3.280310	2.372028	-0.740275
51	7	0	-3.101717	0.112066	-0.749619
52	6	0	-4.512676	0.018942	-1.103792
53	1	0	-4.645327	-0.915395	-1.650532
54	1	0	-4.740346	0.872064	-1.744151
55	6	0	-5.372532	0.036811	0.124959
56	6	0	-5.883360	1.193774	0.773886
57	6	0	-5.814447	-1.098837	0.854589
58	1	0	-5.719748	2.211733	0.442104
59	6	0	-6.636080	0.775825	1.906131
60	1	0	-5.595261	-2.126908	0.593194
61	6	0	-6.594110	-0.649457	1.954871
62	1	0	-7.146182	1.424445	2.606654
63	1	0	-7.068745	-1.277892	2.697504
64	6	0	-9.129116	-1.237130	-0.359897
65	6	0	-8.403156	-0.761322	-1.486437
66	1	0	-7.904855	-1.374897	-2.225788
67	6	0	-8.466128	0.663060	-1.475894
68	1	0	-8.026279	1.329421	-2.206539
69	6	0	-9.640256	-0.117265	0.339985
70	1	0	-10.207601	-0.150063	1.261779
71	6	0	-9.230306	1.055371	-0.341351
72	1	0	-9.447543	2.072261	-0.040004
73	1	0	-9.252213	-2.273825	-0.073000
74	26	0	-7.510730	-0.014553	0.222099
75	1	0	6.181766	0.204023	0.811014
76	1	0	11.201708	0.348188	-2.255214

❖ **1-H^{+}-Fe^{3+}**

Center Number	Atomic Number	Atomic Type	Coordinates (Angstroms) X	Y	Z
1	6	0	8.406971	-0.920862	0.145359
2	6	0	8.761359	-1.616973	-1.009539
3	6	0	9.060599	0.284302	0.441674
4	6	0	9.761263	-1.123881	-1.832197
5	1	0	8.297910	-2.571050	-1.247932
6	6	0	10.040471	0.800859	-0.400923
7	1	0	8.805824	0.828463	1.347342
8	6	0	10.429883	0.077788	-1.565236
9	1	0	10.079017	-1.686522	-2.705736
10	8	0	10.647884	1.985491	-0.169445
11	6	0	10.287414	2.711502	0.990334
12	1	0	9.223028	2.975962	0.976970
13	1	0	10.521527	2.148958	1.901459
14	1	0	10.886331	3.621024	0.961751
15	6	0	7.377811	-1.454383	1.075473
16	1	0	7.614659	-1.252663	2.124401
17	1	0	7.218265	-2.528457	0.950344
18	6	0	5.434508	-1.187811	-0.493764
19	6	0	5.044138	-1.090888	1.948672
20	6	0	4.095773	-0.499271	-0.699198
21	1	0	5.315955	-2.274054	-0.477159
22	1	0	6.170534	-0.907251	-1.250778
23	6	0	3.711858	-0.410553	1.676869
24	1	0	4.929539	-2.176716	1.988777
25	1	0	5.499123	-0.734866	2.876249
26	1	0	3.673123	-0.856857	-1.641650
27	1	0	4.257438	0.589602	-0.809594
28	1	0	3.012492	-0.705900	2.463470
29	1	0	3.842019	0.685461	1.754215
30	7	0	6.013800	-0.807304	0.836960
31	7	0	3.210290	-0.843099	0.392023
32	6	0	1.799243	-0.824354	0.164303
33	6	0	1.059001	0.385666	0.006237
34	6	0	1.156313	-2.043247	0.100313
35	6	0	1.639545	1.676125	0.061807
36	6	0	-0.344825	0.291585	-0.222283
37	6	0	-0.228052	-2.126949	-0.121032
38	1	0	1.743845	-2.946710	0.224837
39	6	0	0.876360	2.810868	-0.093485
40	1	0	2.706533	1.773948	0.229864
41	6	0	-1.107163	1.473084	-0.382117
42	6	0	-0.968587	-0.976567	-0.283284
43	1	0	-0.729766	-3.087879	-0.171225
44	6	0	-0.507559	2.713153	-0.315310
45	1	0	1.342469	3.789479	-0.046545

46	1	0	-1.120908	3.599465	-0.441424
47	6	0	-2.420580	-1.087112	-0.527396
48	6	0	-2.559725	1.395979	-0.630036
49	8	0	-3.022458	-2.145874	-0.551349
50	8	0	-3.280310	2.372028	-0.740275
51	7	0	-3.101717	0.112066	-0.749619
52	6	0	-4.512676	0.018942	-1.103792
53	1	0	-4.645327	-0.915395	-1.650532
54	1	0	-4.740346	0.872064	-1.744151
55	6	0	-5.372532	0.036811	0.124959
56	6	0	-5.883360	1.193774	0.773886
57	6	0	-5.814447	-1.098837	0.854589
58	1	0	-5.719748	2.211733	0.442104
59	6	0	-6.636080	0.775825	1.906131
60	1	0	-5.595261	-2.126908	0.593194
61	6	0	-6.594110	-0.649457	1.954871
62	1	0	-7.146182	1.424445	2.606654
63	1	0	-7.068745	-1.277892	2.697504
64	6	0	-9.129116	-1.237130	-0.359897
65	6	0	-8.403156	-0.761322	-1.486437
66	1	0	-7.904855	-1.374897	-2.225788
67	6	0	-8.466128	0.663060	-1.475894
68	1	0	-8.026279	1.329421	-2.206539
69	6	0	-9.640256	-0.117265	0.339985
70	1	0	-10.207601	-0.150063	1.261779
71	6	0	-9.230306	1.055371	-0.341351
72	1	0	-9.447543	2.072261	-0.040004
73	1	0	-9.252213	-2.273825	-0.073000
74	26	0	-7.510730	-0.014553	0.222099
75	1	0	6.181766	0.204023	0.811014
76	1	0	11.201708	0.348188	-2.255214

❖ **1-Co^{2+}**

Center Number	Atomic Number	Atomic Type	Coordinates (Angstroms) X	Y	Z
1	6	0	7.497229	-1.669546	0.808555
2	6	0	7.871057	-2.992105	0.598458
3	6	0	8.381968	-0.658143	0.418575
4	6	0	9.116362	-3.297131	0.053688
5	1	0	7.199619	-3.798103	0.884964
6	6	0	9.603364	-0.954134	-0.179528
7	1	0	8.066527	0.371554	0.545719
8	6	0	10.017044	-2.303075	-0.346905
9	1	0	9.425603	-4.331481	-0.065293
10	8	0	10.449129	0.003476	-0.651585
11	6	0	10.085666	1.357545	-0.476465
12	1	0	9.150220	1.594727	-0.997477

13	1	0	9.979282	1.614034	0.584306
14	1	0	10.896028	1.945452	-0.908795
15	6	0	6.191090	-1.317904	1.477395
16	1	0	6.381668	-1.034509	2.521638
17	1	0	5.547781	-2.217601	1.513095
18	6	0	5.039909	-0.517464	-0.487747
19	6	0	4.408675	0.291692	1.659320
20	6	0	4.425245	0.721518	-1.125074
21	1	0	4.300663	-1.341747	-0.461544
22	1	0	5.887155	-0.852921	-1.093955
23	6	0	3.793398	1.546210	1.047521
24	1	0	3.618169	-0.475589	1.777344
25	1	0	4.780624	0.536673	2.660637
26	1	0	4.062014	0.512137	-2.132805
27	1	0	5.208777	1.486026	-1.210158
28	1	0	2.949837	1.861631	1.660995
29	1	0	4.538599	2.351679	1.041652
30	7	0	5.513348	-0.193150	0.849352
31	7	0	3.352633	1.299104	-0.326507
32	6	0	2.031451	1.026680	-0.612792
33	6	0	0.998707	1.938089	-0.175047
34	6	0	1.649117	-0.083116	-1.372353
35	6	0	1.279591	3.207103	0.380604
36	6	0	-0.365896	1.591336	-0.387818
37	6	0	0.307082	-0.380387	-1.601324
38	1	0	2.400828	-0.773484	-1.736012
39	6	0	0.269397	4.049375	0.800098
40	1	0	2.309180	3.541120	0.445947
41	6	0	-1.386604	2.457567	0.070327
42	6	0	-0.706779	0.415180	-1.093776
43	1	0	0.037355	-1.274237	-2.155130
44	6	0	-1.070722	3.661192	0.672230
45	1	0	0.513870	5.021069	1.216431
46	1	0	-1.877024	4.304574	1.009011
47	6	0	-2.104613	0.045509	-1.310666
48	6	0	-2.805432	2.095918	-0.110730
49	8	0	-2.447704	-0.968713	-1.909772
50	8	0	-3.721492	2.793219	0.309936
51	7	0	-3.072483	0.916485	-0.797522
52	6	0	-4.480833	0.550690	-0.990076
53	1	0	-4.538523	0.001777	-1.929882
54	1	0	-5.042219	1.481078	-1.076071
55	6	0	-5.011567	-0.280918	0.136092
56	6	0	-5.632883	0.205769	1.327531
57	6	0	-4.962477	-1.706286	0.227976
58	1	0	-5.820859	1.247340	1.551766
59	6	0	-5.977301	-0.911923	2.139010
60	1	0	-4.553544	-2.365340	-0.526278
61	6	0	-5.562499	-2.094481	1.458995
62	1	0	-6.485970	-0.873011	3.093471

63	1	0	-5.701470	-3.109776	1.806979
64	6	0	-7.835614	-1.835343	-1.266050
65	6	0	-7.905517	-0.411992	-1.294335
66	1	0	-7.525262	0.227969	-2.080086
67	6	0	-8.540458	0.021866	-0.094282
68	1	0	-8.726385	1.048968	0.191244
69	6	0	-8.426185	-2.280832	-0.047978
70	1	0	-8.510859	-3.308925	0.279017
71	6	0	-8.861719	-1.133136	0.676089
72	1	0	-9.334904	-1.136855	1.649306
73	1	0	-7.392734	-2.465593	-2.026102
74	1	0	10.953147	-2.577143	-0.786788
75	27	0	-6.869136	-1.041643	0.326291

❖ **<u>1-Co^{3+}</u>**

Center Number	Atomic Number	Atomic Type	Coordinates (Angstroms) X	Y	Z
1	6	0	7.497229	-1.669546	0.808555
2	6	0	7.871057	-2.992105	0.598458
3	6	0	8.381968	-0.658143	0.418575
4	6	0	9.116362	-3.297131	0.053688
5	1	0	7.199619	-3.798103	0.884964
6	6	0	9.603364	-0.954134	-0.179528
7	1	0	8.066527	0.371554	0.545719
8	6	0	10.017044	-2.303075	-0.346905
9	1	0	9.425603	-4.331481	-0.065293
10	8	0	10.449129	0.003476	-0.651585
11	6	0	10.085666	1.357545	-0.476465
12	1	0	9.150220	1.594727	-0.997477
13	1	0	9.979282	1.614034	0.584306
14	1	0	10.896028	1.945452	-0.908795
15	6	0	6.191090	-1.317904	1.477395
16	1	0	6.381668	-1.034509	2.521638
17	1	0	5.547781	-2.217601	1.513095
18	6	0	5.039909	-0.517464	-0.487747
19	6	0	4.408675	0.291692	1.659320
20	6	0	4.425245	0.721518	-1.125074
21	1	0	4.300663	-1.341747	-0.461544
22	1	0	5.887155	-0.852921	-1.093955
23	6	0	3.793398	1.546210	1.047521
24	1	0	3.618169	-0.475589	1.777344
25	1	0	4.780624	0.536673	2.660637
26	1	0	4.062014	0.512137	-2.132805
27	1	0	5.208777	1.486026	-1.210158
28	1	0	2.949837	1.861631	1.660995
29	1	0	4.538599	2.351679	1.041652
30	7	0	5.513348	-0.193150	0.849352

31	7	0	3.352633	1.299104	-0.326507
32	6	0	2.031451	1.026680	-0.612792
33	6	0	0.998707	1.938089	-0.175047
34	6	0	1.649117	-0.083116	-1.372353
35	6	0	1.279591	3.207103	0.380604
36	6	0	-0.365896	1.591336	-0.387818
37	6	0	0.307082	-0.380387	-1.601324
38	1	0	2.400828	-0.773484	-1.736012
39	6	0	0.269397	4.049375	0.800098
40	1	0	2.309180	3.541120	0.445947
41	6	0	-1.386604	2.457567	0.070327
42	6	0	-0.706779	0.415180	-1.093776
43	1	0	0.037355	-1.274237	-2.155130
44	6	0	-1.070722	3.661192	0.672230
45	1	0	0.513870	5.021069	1.216431
46	1	0	-1.877024	4.304574	1.009011
47	6	0	-2.104613	0.045509	-1.310666
48	6	0	-2.805432	2.095918	-0.110730
49	8	0	-2.447704	-0.968713	-1.909772
50	8	0	-3.721492	2.793219	0.309936
51	7	0	-3.072483	0.916485	-0.797522
52	6	0	-4.480833	0.550690	-0.990076
53	1	0	-4.538523	0.001777	-1.929882
54	1	0	-5.042219	1.481078	-1.076071
55	6	0	-5.011567	-0.280918	0.136092
56	6	0	-5.632883	0.205769	1.327531
57	6	0	-4.962477	-1.706286	0.227976
58	1	0	-5.820859	1.247340	1.551766
59	6	0	-5.977301	-0.911923	2.139010
60	1	0	-4.553544	-2.365340	-0.526278
61	6	0	-5.562499	-2.094481	1.458995
62	1	0	-6.485970	-0.873011	3.093471
63	1	0	-5.701470	-3.109776	1.806979
64	6	0	-7.835614	-1.835343	-1.266050
65	6	0	-7.905517	-0.411992	-1.294335
66	1	0	-7.525262	0.227969	-2.080086
67	6	0	-8.540458	0.021866	-0.094282
68	1	0	-8.726385	1.048968	0.191244
69	6	0	-8.426185	-2.280832	-0.047978
70	1	0	-8.510859	-3.308925	0.279017
71	6	0	-8.861719	-1.133136	0.676089
72	1	0	-9.334904	-1.136855	1.649306
73	1	0	-7.392734	-2.465593	-2.026102
74	1	0	10.953147	-2.577143	-0.786788
75	27	0	-6.869136	-1.041643	0.326291

❖ **1-H^+-Co^{2+}**

Center Atomic Atomic Coordinates (Angstroms)

Number	Number	Type	X	Y	Z
1	6	0	8.392450	-0.915947	0.246137
2	6	0	8.822745	-1.605463	-0.884022
3	6	0	8.969566	0.329007	0.534034
4	6	0	9.820858	-1.075742	-1.693147
5	1	0	8.405696	-2.580851	-1.120900
6	6	0	9.934397	0.886864	-0.296888
7	1	0	8.639350	0.878190	1.410024
8	6	0	10.411934	0.170182	-1.433024
9	1	0	10.192829	-1.647863	-2.536874
10	8	0	10.452969	2.125887	-0.099154
11	6	0	10.026642	2.861720	1.031110
12	1	0	8.948986	3.060639	0.996895
13	1	0	10.270101	2.342224	1.965237
14	1	0	10.567787	3.807249	0.994384
15	6	0	7.331258	-1.471503	1.139857
16	1	0	7.532910	-1.260134	2.192735
17	1	0	7.212159	-2.549963	1.016273
18	6	0	5.444768	-1.219274	-0.495528
19	6	0	4.973631	-1.231113	1.923463
20	6	0	4.097715	-0.561742	-0.731657
21	1	0	5.355793	-2.308123	-0.519617
22	1	0	6.185827	-0.894113	-1.227124
23	6	0	3.632504	-0.578648	1.641232
24	1	0	4.889257	-2.319963	1.911031
25	1	0	5.379284	-0.903790	2.882619
26	1	0	3.721355	-0.909488	-1.696770
27	1	0	4.233025	0.531403	-0.809922
28	1	0	2.921635	-0.939168	2.388787
29	1	0	3.722903	0.513095	1.777273
30	7	0	5.966913	-0.865041	0.863087
31	7	0	3.179916	-0.962311	0.317921
32	6	0	1.782027	-0.902131	0.049568
33	6	0	1.061267	0.325624	-0.084536
34	6	0	1.113876	-2.104230	-0.077390
35	6	0	1.662107	1.603812	0.030177
36	6	0	-0.338729	0.264924	-0.346502
37	6	0	-0.265139	-2.152633	-0.334533
38	1	0	1.684220	-3.020859	0.028894
39	6	0	0.919345	2.755390	-0.107046
40	1	0	2.724284	1.681621	0.229466
41	6	0	-1.081738	1.460604	-0.481783
42	6	0	-0.986740	-0.985371	-0.467921
43	1	0	-0.779060	-3.103311	-0.430297
44	6	0	-0.459879	2.686916	-0.363544
45	1	0	1.401289	3.723120	-0.015621
46	1	0	-1.054156	3.588179	-0.471376
47	6	0	-2.437239	-1.057059	-0.733863
48	6	0	-2.534158	1.413063	-0.746811

49	8	0	-3.034173	-2.119396	-0.837366
50	8	0	-3.213986	2.423320	-0.860267
51	7	0	-3.114314	0.152430	-0.869025
52	6	0	-4.559941	0.093747	-1.127857
53	1	0	-4.739145	-0.803754	-1.719721
54	1	0	-4.810780	0.974431	-1.718837
55	6	0	-5.361557	0.060496	0.135758
56	6	0	-5.837174	1.194732	0.864234
57	6	0	-5.766648	-1.110232	0.848795
58	1	0	-5.695237	2.227130	0.573497
59	6	0	-6.542149	0.725051	2.008025
60	1	0	-5.562428	-2.128101	0.544276
61	6	0	-6.498892	-0.700000	1.998298
62	1	0	-7.044252	1.340648	2.742935
63	1	0	-6.962353	-1.354822	2.724640
64	6	0	-8.519792	-1.188646	-0.860396
65	6	0	-8.097422	-0.033092	-1.579909
66	1	0	-7.507277	-0.029137	-2.487279
67	6	0	-8.566061	1.117111	-0.880723
68	1	0	-8.394035	2.147324	-1.163698
69	6	0	-9.248439	-0.752526	0.283945
70	1	0	-9.685701	-1.390794	1.040560
71	6	0	-9.277081	0.672313	0.271361
72	1	0	-9.740041	1.305683	1.016716
73	1	0	-8.306272	-2.215930	-1.125209
74	1	0	6.091033	0.152693	0.888823
75	1	0	11.163477	0.558945	-2.087964
76	27	0	-7.368089	-0.001618	0.308262

❖ **1-H^{+}-Co^{3+}**

Center Number	Atomic Number	Atomic Type	Coordinates (Angstroms) X	Y	Z
1	6	0	8.392450	-0.915947	0.246137
2	6	0	8.822745	-1.605463	-0.884022
3	6	0	8.969566	0.329007	0.534034
4	6	0	9.820858	-1.075742	-1.693147
5	1	0	8.405696	-2.580851	-1.120900
6	6	0	9.934397	0.886864	-0.296888
7	1	0	8.639350	0.878190	1.410024
8	6	0	10.411934	0.170182	-1.433024
9	1	0	10.192829	-1.647863	-2.536874
10	8	0	10.452969	2.125887	-0.099154
11	6	0	10.026642	2.861720	1.031110
12	1	0	8.948986	3.060639	0.996895
13	1	0	10.270101	2.342224	1.965237
14	1	0	10.567787	3.807249	0.994384

15	6	0	7.331258	-1.471503	1.139857
16	1	0	7.532910	-1.260134	2.192735
17	1	0	7.212159	-2.549963	1.016273
18	6	0	5.444768	-1.219274	-0.495528
19	6	0	4.973631	-1.231113	1.923463
20	6	0	4.097715	-0.561742	-0.731657
21	1	0	5.355793	-2.308123	-0.519617
22	1	0	6.185827	-0.894113	-1.227124
23	6	0	3.632504	-0.578648	1.641232
24	1	0	4.889257	-2.319963	1.911031
25	1	0	5.379284	-0.903790	2.882619
26	1	0	3.721355	-0.909488	-1.696770
27	1	0	4.233025	0.531403	-0.809922
28	1	0	2.921635	-0.939168	2.388787
29	1	0	3.722903	0.513095	1.777273
30	7	0	5.966913	-0.865041	0.863087
31	7	0	3.179916	-0.962311	0.317921
32	6	0	1.782027	-0.902131	0.049568
33	6	0	1.061267	0.325624	-0.084536
34	6	0	1.113876	-2.104230	-0.077390
35	6	0	1.662107	1.603812	0.030177
36	6	0	-0.338729	0.264924	-0.346502
37	6	0	-0.265139	-2.152633	-0.334533
38	1	0	1.684220	-3.020859	0.028894
39	6	0	0.919345	2.755390	-0.107046
40	1	0	2.724284	1.681621	0.229466
41	6	0	-1.081738	1.460604	-0.481783
42	6	0	-0.986740	-0.985371	-0.467921
43	1	0	-0.779060	-3.103311	-0.430297
44	6	0	-0.459879	2.686916	-0.363544
45	1	0	1.401289	3.723120	-0.015621
46	1	0	-1.054156	3.588179	-0.471376
47	6	0	-2.437239	-1.057059	-0.733863
48	6	0	-2.534158	1.413063	-0.746811
49	8	0	-3.034173	-2.119396	-0.837366
50	8	0	-3.213986	2.423320	-0.860267
51	7	0	-3.114314	0.152430	-0.869025
52	6	0	-4.559941	0.093747	-1.127857
53	1	0	-4.739145	-0.803754	-1.719721
54	1	0	-4.810780	0.974431	-1.718837
55	6	0	-5.361557	0.060496	0.135758
56	6	0	-5.837174	1.194732	0.864234
57	6	0	-5.766648	-1.110232	0.848795
58	1	0	-5.695237	2.227130	0.573497
59	6	0	-6.542149	0.725051	2.008025
60	1	0	-5.562428	-2.128101	0.544276
61	6	0	-6.498892	-0.700000	1.998298
62	1	0	-7.044252	1.340648	2.742935
63	1	0	-6.962353	-1.354822	2.724640
64	6	0	-8.519792	-1.188646	-0.860396

65	6	0	-8.097422	-0.033092	-1.579909
66	1	0	-7.507277	-0.029137	-2.487279
67	6	0	-8.566061	1.117111	-0.880723
68	1	0	-8.394035	2.147324	-1.163698
69	6	0	-9.248439	-0.752526	0.283945
70	1	0	-9.685701	-1.390794	1.040560
71	6	0	-9.277081	0.672313	0.271361
72	1	0	-9.740041	1.305683	1.016716
73	1	0	-8.306272	-2.215930	-1.125209
74	1	0	6.091033	0.152693	0.888823
75	1	0	11.163477	0.558945	-2.087964
76	27	0	-7.368089	-0.001618	0.308262

❖ **1-Ni^{2+}**

Center Number	Atomic Number	Atomic Type	Coordinates (Angstroms) X	Y	Z
1	6	0	8.434957	-0.914923	0.252805
2	6	0	8.866003	-1.603932	-0.877376
3	6	0	9.011800	0.329955	0.541581
4	6	0	9.864576	-1.073796	-1.685662
5	1	0	8.449175	-2.579253	-1.114921
6	6	0	9.977103	0.888233	-0.288511
7	1	0	8.681001	0.878745	1.417596
8	6	0	10.455396	0.172063	-1.424651
9	1	0	10.237111	-1.645536	-2.529398
10	8	0	10.495458	2.127212	-0.089938
11	6	0	10.068376	2.862539	1.040371
12	1	0	8.990725	3.061391	1.005573
13	1	0	10.311297	2.342670	1.974431
14	1	0	10.609472	3.808124	1.004376
15	6	0	7.373255	-1.470933	1.145637
16	1	0	7.574240	-1.259990	2.198727
17	1	0	7.254314	-2.549350	1.021527
18	6	0	5.487757	-1.218162	-0.490808
19	6	0	5.015126	-1.231051	1.927885
20	6	0	4.140800	-0.560633	-0.727495
21	1	0	5.398879	-2.307008	-0.515409
22	1	0	6.229243	-0.892638	-1.221810
23	6	0	3.674123	-0.578569	1.645099
24	1	0	4.930842	-2.319901	1.914945
25	1	0	5.420160	-0.904099	2.887429
26	1	0	3.765063	-0.908004	-1.692986
27	1	0	4.276075	0.532554	-0.805219
28	1	0	2.962820	-0.939456	2.392063
29	1	0	3.764356	0.513123	1.781653
30	7	0	6.009035	-0.864459	0.868277
31	7	0	3.222383	-0.961712	0.321347

32	6	0	1.824655	-0.901525	0.052156
33	6	0	1.103886	0.326231	-0.081880
34	6	0	1.156675	-2.103623	-0.075719
35	6	0	1.704557	1.604416	0.033741
36	6	0	-0.295944	0.265534	-0.344737
37	6	0	-0.222177	-2.152022	-0.333735
38	1	0	1.727023	-3.020252	0.030534
39	6	0	0.961792	2.755995	-0.103459
40	1	0	2.766604	1.682222	0.233719
41	6	0	-1.038960	1.461215	-0.479976
42	6	0	-0.943785	-0.984759	-0.467080
43	1	0	-0.735966	-3.102698	-0.430214
44	6	0	-0.417268	2.687524	-0.360839
45	1	0	1.443606	3.723724	-0.011332
46	1	0	-1.011546	3.588787	-0.468661
47	6	0	-2.394113	-1.056445	-0.733949
48	6	0	-2.491213	1.413675	-0.745922
49	8	0	-2.990903	-2.118785	-0.838266
50	8	0	-3.171047	2.423927	-0.859375
51	7	0	-3.071197	0.153049	-0.869022
52	6	0	-4.516659	0.094365	-1.128773
53	1	0	-4.695429	-0.802902	-1.721123
54	1	0	-4.767199	0.975276	-1.719539
55	6	0	-5.319053	0.060523	0.134333
56	6	0	-5.795207	1.194417	0.862990
57	6	0	-5.724496	-1.110535	0.846629
58	1	0	-5.653169	2.226948	0.572773
59	6	0	-6.500853	0.724204	2.006147
60	1	0	-5.520011	-2.128260	0.541810
61	6	0	-6.457482	-0.700839	1.995850
62	1	0	-7.003458	1.339455	2.741004
63	1	0	-6.921342	-1.356001	2.721631
64	6	0	-8.476577	-1.188442	-0.864297
65	6	0	-8.053850	-0.032554	-1.583065
66	1	0	-7.463145	-0.028174	-2.490068
67	6	0	-8.523009	1.117321	-0.883686
68	1	0	-8.350887	2.147665	-1.166124
69	6	0	-9.205965	-0.752856	0.279776
70	1	0	-9.643646	-1.391474	1.035852
71	6	0	-9.234707	0.671986	0.267771
72	1	0	-9.698176	1.305009	1.013105
73	1	0	-8.262815	-2.215597	-1.129408
74	1	0	11.207314	0.561157	-2.078964
75	28	0	-7.325687	-0.001815	0.305570

❖ **1-Ni^{3+}**

Center Number	Atomic Number	Atomic Type	Coordinates (Angstroms) X	Y	Z

1	6	0	8.434957	-0.914923	0.252805
2	6	0	8.866003	-1.603932	-0.877376
3	6	0	9.011800	0.329955	0.541581
4	6	0	9.864576	-1.073796	-1.685662
5	1	0	8.449175	-2.579253	-1.114921
6	6	0	9.977103	0.888233	-0.288511
7	1	0	8.681001	0.878745	1.417596
8	6	0	10.455396	0.172063	-1.424651
9	1	0	10.237111	-1.645536	-2.529398
10	8	0	10.495458	2.127212	-0.089938
11	6	0	10.068376	2.862539	1.040371
12	1	0	8.990725	3.061391	1.005573
13	1	0	10.311297	2.342670	1.974431
14	1	0	10.609472	3.808124	1.004376
15	6	0	7.373255	-1.470933	1.145637
16	1	0	7.574240	-1.259990	2.198727
17	1	0	7.254314	-2.549350	1.021527
18	6	0	5.487757	-1.218162	-0.490808
19	6	0	5.015126	-1.231051	1.927885
20	6	0	4.140800	-0.560633	-0.727495
21	1	0	5.398879	-2.307008	-0.515409
22	1	0	6.229243	-0.892638	-1.221810
23	6	0	3.674123	-0.578569	1.645099
24	1	0	4.930842	-2.319901	1.914945
25	1	0	5.420160	-0.904099	2.887429
26	1	0	3.765063	-0.908004	-1.692986
27	1	0	4.276075	0.532554	-0.805219
28	1	0	2.962820	-0.939456	2.392063
29	1	0	3.764356	0.513123	1.781653
30	7	0	6.009035	-0.864459	0.868277
31	7	0	3.222383	-0.961712	0.321347
32	6	0	1.824655	-0.901525	0.052156
33	6	0	1.103886	0.326231	-0.081880
34	6	0	1.156675	-2.103623	-0.075719
35	6	0	1.704557	1.604416	0.033741
36	6	0	-0.295944	0.265534	-0.344737
37	6	0	-0.222177	-2.152022	-0.333735
38	1	0	1.727023	-3.020252	0.030534
39	6	0	0.961792	2.755995	-0.103459
40	1	0	2.766604	1.682222	0.233719
41	6	0	-1.038960	1.461215	-0.479976
42	6	0	-0.943785	-0.984759	-0.467080
43	1	0	-0.735966	-3.102698	-0.430214
44	6	0	-0.417268	2.687524	-0.360839
45	1	0	1.443606	3.723724	-0.011332
46	1	0	-1.011546	3.588787	-0.468661
47	6	0	-2.394113	-1.056445	-0.733949
48	6	0	-2.491213	1.413675	-0.745922
49	8	0	-2.990903	-2.118785	-0.838266

50	8	0	-3.171047	2.423927	-0.859375
51	7	0	-3.071197	0.153049	-0.869022
52	6	0	-4.516659	0.094365	-1.128773
53	1	0	-4.695429	-0.802902	-1.721123
54	1	0	-4.767199	0.975276	-1.719539
55	6	0	-5.319053	0.060523	0.134333
56	6	0	-5.795207	1.194417	0.862990
57	6	0	-5.724496	-1.110535	0.846629
58	1	0	-5.653169	2.226948	0.572773
59	6	0	-6.500853	0.724204	2.006147
60	1	0	-5.520011	-2.128260	0.541810
61	6	0	-6.457482	-0.700839	1.995850
62	1	0	-7.003458	1.339455	2.741004
63	1	0	-6.921342	-1.356001	2.721631
64	6	0	-8.476577	-1.188442	-0.864297
65	6	0	-8.053850	-0.032554	-1.583065
66	1	0	-7.463145	-0.028174	-2.490068
67	6	0	-8.523009	1.117321	-0.883686
68	1	0	-8.350887	2.147665	-1.166124
69	6	0	-9.205965	-0.752856	0.279776
70	1	0	-9.643646	-1.391474	1.035852
71	6	0	-9.234707	0.671986	0.267771
72	1	0	-9.698176	1.305009	1.013105
73	1	0	-8.262815	-2.215597	-1.129408
74	1	0	11.207314	0.561157	-2.078964
75	28	0	-7.325687	-0.001815	0.305570

❖ **$1\text{-}H^{+}\text{-}Ni^{2+}$**

Center Number	Atomic Number	Atomic Type	Coordinates (Angstroms) X	Y	Z
1	6	0	8.415648	-0.915951	0.246686
2	6	0	8.846147	-1.605465	-0.883395
3	6	0	8.992713	0.329002	0.534690
4	6	0	9.844407	-1.075743	-1.692339
5	1	0	8.429140	-2.580853	-1.120351
6	6	0	9.957695	0.886860	-0.296057
7	1	0	8.662339	0.878184	1.410621
8	6	0	10.435437	0.170179	-1.432107
9	1	0	10.216530	-1.647864	-2.535999
10	8	0	10.476233	2.125882	-0.098227
11	6	0	10.049701	2.861713	1.031961
12	1	0	8.972051	3.060634	0.997551
13	1	0	10.292991	2.342216	1.966132
14	1	0	10.590854	3.807242	0.995335
15	6	0	7.354293	-1.471507	1.140214
16	1	0	7.555755	-1.260140	2.193128
17	1	0	7.235215	-2.549966	1.016606

18	6	0	5.468099	-1.219273	-0.495512
19	6	0	4.996525	-1.231116	1.923393
20	6	0	4.121090	-0.561740	-0.731884
21	1	0	5.379128	-2.308122	-0.519619
22	1	0	6.209291	-0.894112	-1.226973
23	6	0	3.655449	-0.578649	1.640921
24	1	0	4.912152	-2.319965	1.910944
25	1	0	5.402004	-0.903795	2.882623
26	1	0	3.744904	-0.909484	-1.697066
27	1	0	4.256415	0.531405	-0.810123
28	1	0	2.944445	-0.939169	2.388346
29	1	0	3.745825	0.513093	1.776979
30	7	0	5.989999	-0.865043	0.863198
31	7	0	3.203101	-0.962310	0.317527
32	6	0	1.805260	-0.902127	0.048921
33	6	0	1.084526	0.325628	-0.085312
34	6	0	1.137131	-2.104226	-0.078159
35	6	0	1.685346	1.603816	0.029513
36	6	0	-0.315423	0.264930	-0.347531
37	6	0	-0.241837	-2.152627	-0.335552
38	1	0	1.707455	-3.020856	0.028226
39	6	0	0.942610	2.755395	-0.107843
40	1	0	2.747487	1.681623	0.228994
41	6	0	-1.058406	1.460611	-0.482945
42	6	0	-0.963413	-0.985364	-0.469069
43	1	0	-0.755741	-3.103304	-0.431411
44	6	0	-0.436567	2.686922	-0.364591
45	1	0	1.424539	3.723124	-0.016330
46	1	0	-1.030823	3.588186	-0.472530
47	6	0	-2.413863	-1.057050	-0.735274
48	6	0	-2.510778	1.413072	-0.748236
49	8	0	-3.010780	-2.119387	-0.838887
50	8	0	-3.190585	2.423330	-0.861813
51	7	0	-3.090914	0.152440	-0.870556
52	6	0	-4.536494	0.093758	-1.129651
53	1	0	-4.715591	-0.803741	-1.721548
54	1	0	-4.787224	0.974443	-1.720674
55	6	0	-5.338338	0.060506	0.133820
56	6	0	-5.814085	1.194741	0.862211
57	6	0	-5.743559	-1.110223	0.846781
58	1	0	-5.672095	2.227140	0.571502
59	6	0	-6.519268	0.725060	2.005873
60	1	0	-5.539285	-2.128091	0.542298
61	6	0	-6.476011	-0.699992	1.996152
62	1	0	-7.021504	1.340656	2.740693
63	1	0	-6.939604	-1.354815	2.722409
64	6	0	-8.496394	-1.188632	-0.862908
65	6	0	-8.073893	-0.033077	-1.582343
66	1	0	-7.483583	-0.029121	-2.489606
67	6	0	-8.542657	1.117126	-0.883240

68	1	0	-8.370579	2.147339	-1.166183
69	6	0	-9.225248	-0.752512	0.281301
70	1	0	-9.662647	-1.390781	1.037836
71	6	0	-9.253886	0.672327	0.268714
72	1	0	-9.716980	1.305696	1.013986
73	1	0	-8.282827	-2.215915	-1.127684
74	1	0	6.114115	0.152691	0.888957
75	1	0	11.187098	0.558942	-2.086911
76	28	0	-7.344902	-0.001606	0.305960

❖ **$1\text{-}H^{+}\text{-}Ni^{3+}$**

Center Number	Atomic Number	Atomic Type	Coordinates (Angstroms) X	Y	Z
1	6	0	8.415648	-0.915951	0.246686
2	6	0	8.846147	-1.605465	-0.883395
3	6	0	8.992713	0.329002	0.534690
4	6	0	9.844407	-1.075743	-1.692339
5	1	0	8.429140	-2.580853	-1.120351
6	6	0	9.957695	0.886860	-0.296057
7	1	0	8.662339	0.878184	1.410621
8	6	0	10.435437	0.170179	-1.432107
9	1	0	10.216530	-1.647864	-2.535999
10	8	0	10.476233	2.125882	-0.098227
11	6	0	10.049701	2.861713	1.031961
12	1	0	8.972051	3.060634	0.997551
13	1	0	10.292991	2.342216	1.966132
14	1	0	10.590854	3.807242	0.995335
15	6	0	7.354293	-1.471507	1.140214
16	1	0	7.555755	-1.260140	2.193128
17	1	0	7.235215	-2.549966	1.016606
18	6	0	5.468099	-1.219273	-0.495512
19	6	0	4.996525	-1.231116	1.923393
20	6	0	4.121090	-0.561740	-0.731884
21	1	0	5.379128	-2.308122	-0.519619
22	1	0	6.209291	-0.894112	-1.226973
23	6	0	3.655449	-0.578649	1.640921
24	1	0	4.912152	-2.319965	1.910944
25	1	0	5.402004	-0.903795	2.882623
26	1	0	3.744904	-0.909484	-1.697066
27	1	0	4.256415	0.531405	-0.810123
28	1	0	2.944445	-0.939169	2.388346
29	1	0	3.745825	0.513093	1.776979
30	7	0	5.989999	-0.865043	0.863198
31	7	0	3.203101	-0.962310	0.317527
32	6	0	1.805260	-0.902127	0.048921
33	6	0	1.084526	0.325628	-0.085312
34	6	0	1.137131	-2.104226	-0.078159

35	6	0	1.685346	1.603816	0.029513
36	6	0	-0.315423	0.264930	-0.347531
37	6	0	-0.241837	-2.152627	-0.335552
38	1	0	1.707455	-3.020856	0.028226
39	6	0	0.942610	2.755395	-0.107843
40	1	0	2.747487	1.681623	0.228994
41	6	0	-1.058406	1.460611	-0.482945
42	6	0	-0.963413	-0.985364	-0.469069
43	1	0	-0.755741	-3.103304	-0.431411
44	6	0	-0.436567	2.686922	-0.364591
45	1	0	1.424539	3.723124	-0.016330
46	1	0	-1.030823	3.588186	-0.472530
47	6	0	-2.413863	-1.057050	-0.735274
48	6	0	-2.510778	1.413072	-0.748236
49	8	0	-3.010780	-2.119387	-0.838887
50	8	0	-3.190585	2.423330	-0.861813
51	7	0	-3.090914	0.152440	-0.870556
52	6	0	-4.536494	0.093758	-1.129651
53	1	0	-4.715591	-0.803741	-1.721548
54	1	0	-4.787224	0.974443	-1.720674
55	6	0	-5.338338	0.060506	0.133820
56	6	0	-5.814085	1.194741	0.862211
57	6	0	-5.743559	-1.110223	0.846781
58	1	0	-5.672095	2.227140	0.571502
59	6	0	-6.519268	0.725060	2.005873
60	1	0	-5.539285	-2.128091	0.542298
61	6	0	-6.476011	-0.699992	1.996152
62	1	0	-7.021504	1.340656	2.740693
63	1	0	-6.939604	-1.354815	2.722409
64	6	0	-8.496394	-1.188632	-0.862908
65	6	0	-8.073893	-0.033077	-1.582343
66	1	0	-7.483583	-0.029121	-2.489606
67	6	0	-8.542657	1.117126	-0.883240
68	1	0	-8.370579	2.147339	-1.166183
69	6	0	-9.225248	-0.752512	0.281301
70	1	0	-9.662647	-1.390781	1.037836
71	6	0	-9.253886	0.672327	0.268714
72	1	0	-9.716980	1.305696	1.013986
73	1	0	-8.282827	-2.215915	-1.127684
74	1	0	6.114115	0.152691	0.888957
75	1	0	11.187098	0.558942	-2.086911
76	28	0	-7.344902	-0.001606	0.305960
